# Organic Moiety on Sn(IV) Does Matter for In Vitro Mode of Action: *n*Bu_3_Sn(IV) Compounds with Carboxylato *N*-Functionalized 2-Quinolones Induce Anoikis-like Cell Death in A375 Cells

**DOI:** 10.3390/pharmaceutics16121529

**Published:** 2024-11-28

**Authors:** Marijana P. Kasalović, Sanja Jelača, Dušan Dimić, Danijela Maksimović-Ivanić, Verica V. Jevtić, Sanja Mijatović, Tobias Rüffer, Goran N. Kaluđerović, Nebojša Đ. Pantelić

**Affiliations:** 1 Department of Engineering and Natural Sciences, University of Applied Sciences Merseburg, Eberhard-Leibnitz-Straße 2, 06217 Merseburg, Germany; marijana.kasalovic@pmf.kg.ac.rs; 2 Department of Chemistry, Faculty of Science, University of Kragujevac, Radoja Domanovića 12, 34000 Kragujevac, Serbia; verica.jevtic@pmf.kg.ac.rs; 3 Department of Immunology, Institute for Biological Research “Siniša Stanković”—National Institute of the Republic of Serbia, University of Belgrade, Bulevar Despota Stefana 142, 11108 Belgrade, Serbia; sanja.jelaca@ibiss.bg.ac.rs (S.J.); nelamax@ibiss.bg.ac.rs (D.M.-I.); sanjamama@ibiss.bg.ac.rs (S.M.); 4 Faculty of Physical Chemistry, University of Belgrade, Studentski Trg 12-16, 11000 Belgrade, Serbia; ddimic@ffh.bg.ac.rs; 5 Institute of Chemistry, Chemnitz University of Technology, Straße der Nationen 62, 09111 Chemnitz, Germany; tobias.rueffer@chemie.tu-chemnitz.de; 6 Department of Chemistry and Biochemistry, Faculty of Agriculture, University of Belgrade, Nemanjina 6, 11080 Belgrade, Serbia

**Keywords:** tributyltin(IV) compounds, 2-quinolones, in vitro cytotoxicity, apoptosis, BSA interactions, molecular docking

## Abstract

**Objectives:** New tributyltin(IV) complexes containing the carboxylate ligands 3-(4-methyl-2-oxoquinolin-1(2H)-yl)propanoic acid (**HL1**) and 2-(4-methyl-2-oxoquinolin-1(2H)-yl)acetic acid (**HL2**) have been synthesized. **Methods:** Their structures have been determined by elemental microanalysis, FT-IR and multinuclear NMR (^1^H, ^13^C and ^119^Sn) spectroscopy and X-ray diffraction study. A solution state NMR analysis reveals a four-coordinated tributyltin(IV) complex in non-polar solvents, while an X-Ray crystallographic analysis confirms a five-coordinated trigonal-bipyramidal geometry around the tin atom due to the formation of 1D chains. A theoretical structural analysis was performed by optimization employing B3LYP-D3BJ functional and 6-311++G(d,p)/def2-TZVP(Sn) basis sets for H, C, N, O/Sn, respectively. The interactions between tin(IV) and surrounding atoms were examined by QTAIM approach. The in vitro antiproliferative activity of the synthesized compounds was evaluated by MTT and CV assays versus MCF-7 (human breast adenocarcinoma), HCT116 (human colorectal carcinoma), A375 (human melanoma), 4T1 (mouse breast carcinoma), CT26 (mouse colon carcinoma) and B16 (mouse melanoma) tumor cell lines. **Results:** Both synthesized compounds (***n*Bu_3_SnL1** and ***n*Bu_3_SnL2**) exerted powerful micromolar IC_50_ cytotoxicity values and demonstrated high selectivity toward malignant cells. Both experimental drugs affected cell adhesion and induced anchorage independent apoptosis, a favorable type of cell death with an essential role in cancer dissemination prevention. The BSA-binding affinity of the obtained organotin compounds was followed by spectrofluorometric titration and molecular docking simulations. **Conclusions:** The tributyltin(IV) compounds selectively induce anoikis-like cell death in A375 cells, also highlighting the importance of the organic moiety on the tin(IV) ion in the mechanism of action.

## 1. Introduction

Since cisplatin was successfully introduced as a metal-based chemotherapeutic drug, many researchers have focused their investigations onto the design of new metal-based complexes and organometallic compounds [1,2,3]. Cisplatin, along with its analogues carboplatin and oxaliplatin, is commonly employed in the clinical treatment of a variety of malignant cancers, such as breast, head and neck, ovarian, small-cell lung, and colorectal [4,5]. The demand for the development of new chemotherapeutic drugs is of importance, as the effectiveness of platinum-based medicines is constrained by resistance and can be poisonousness, among other adverse side effects [6,7,8]. A promising treatment strategy for overcoming these disadvantages, is a combination of cisplatin with other drugs for the treatment of many cancers [9]. Many transition metal complexes (Ti, Ga, Pd, Au, Ru, Sn), have already found their place in chemotherapy, providing the minimalization of side effects and resistance associated with platinum-based drugs [10,11,12]. An alternative approach is to examine the anticancer potential of non-platinum transition metal complexes. Among them, organotin(IV) compounds have demonstrated promising cytotoxicity, often surpassing the effectiveness of the standard chemotherapeutical drug, cisplatin, with fewer side effects [13,14].

Organotin(IV) compounds constitute an important class of compounds with a wide horizon for commercial and biological applications [15,16,17]. In recent years, many organotin(IV) compounds have been screened for their therapeutic potential; this increased interest is attributed to their biological properties, such as antibacterial, antimalarial, schizonticidal, antidiabetic, antileishmanial, anti-HCV, anti-hyperbilirubinemia, among others [18]. Because of common tin and platinum properties, particularly oxidation states +2 and +4, tin compounds were among the first for which the anticancer potential was investigated [3,19]. The biological activity is essentially related to the number and nature of the alkyl/aryl substituent attached directly to the tin center, but the nature of the anionic ligand cannot be ignored [20,21]. Depending upon the number of alkyl/aryl substituents, the biological activity decreases in the order R_3_Sn^+^ > R_2_Sn^2+^ > RSn^3+^ [20,22]. With respect to cytotoxic activity, the highest cytotoxicity of triorganotin(IV) compounds can be attributed either to their highest lipophilicity or their affinity to bind to proteins [21,23,24]. Organotin(IV) moiety can easily form complexes with different ligands containing *O*, *S*, *N*, and *P* donor atoms [25,26]. SAR (structural activity relationship) study based on the comprehensive literature demonstrated that all anticancer active organotin(IV) compounds must possess certain properties. The cytotoxic activity arises due to the availability of a coordination position around tin center, and relatively stable ligand-tin bonds with slow hydrolytic decomposition [27,28]. Organotin(IV) carboxylates exert the highest cytotoxicity when compared to thiolato- and dithiocarbamato-analogues, probably because sulfur donor ligands form very stable coordination bonds with organotin(IV) moiety [26,29]. Tributyltin(IV) complexes demonstrated multiple times better cytotoxic activity than clinically used cisplatin [30]. The evaluation of anticancer activity of organotin(IV) complexes is something that is worthy of attention, because they exhibit lower toxicity in comparison to platinum-based drugs, and induce cell death at lower doses, reducing the likelihood of resistance developing in cells [31,32].

An interesting approach for the synthesis of novel organotin(IV) compounds with potential anticancer activity is in combination with ligands that have biologically active pharmacophores. The triorganotin(IV) moiety, with its lipophilic nature, serves as a carrier through the cell membrane, while a ligand plays a major role in directing these compounds to the target cite to determine their bioavailability [1,23]. Due to its great performances, good oral absorption, safety, excellent bioavailability, and the possibility for numerous structural modifications, a quinolone core represents one of the most desirable heterocycles in medicinal chemistry [33,34,35]. Quinolones have proven antimicrobial, antioxidant, antimalarial, antiviral, anti-inflammatory, and anticancer activity [36,37]. The substitutions at different positions of the quinolone ring can provide a panel of novel quinolone derivatives with various pharmacokinetic properties. For example, structural modifications at N-1 position give dual anticancer and antibacterial activities, while modification at positions 2 and 4 significantly affect biological activity [35,38].

Our previous studies have shown that diphenyl-/triphenyl-/trimethyltin(IV) compounds with carboxylato *N*-functionalized 2-quinolone derivatives demonstrate high cytotoxic activity versus various tumor cell lines and represent promising candidates as anticancer drugs [39,40,41]. In that light, we report here two newly prepared tributyltin(IV) complexes bearing *N*-substituted 2-quinolone derivatives in order to confirm the influence of the nature of the substituents bonded to the Sn(IV) center on cytotoxicity. These compounds were fully characterized via FT-IR and multinuclear NMR spectroscopy and confirmed by X-ray crystallographic analysis. The BSA-binding affinities of obtained compounds were investigated using spectrofluorometric titration and simulations through molecular docking. Cytotoxicity was assessed through MTT and CV assays toward various tumor cell lines, including MCF-7 (human breast adenocarcinoma), HCT116 (human colorectal carcinoma), A375 (human melanoma), 4T1 (mouse breast carcinoma), CT26 (mouse colon carcinoma), and B16 (mouse melanoma). In addition, the mechanism of their action was evaluated using flow cytometry.

## 2. Materials and Methods

### 2.1. Instrumentations

Melting point was obtained at Mel-Temp Capillary Melting Point Apparatus, model 1001 (Thomas Scientific, San Francisco, CA, USA). Elemental analysis was carried out by the University of Belgrade (Vario EL III C, H, N and S Elemental Analyzer, Heraeus Group, Hanau, Germany) and the Institute of Chemistry, Chemnitz University of Technology (Heraeus VARIO EL oven; Heraeus Group, Hanau, Germany). Infrared spectra were obtained using a Bruker Vertex 70 with Diamond ATR unit (Bruker, Karlsruhe, Germany), covering the range from 4000 to 200 cm^−1^. ^1^H, ^13^C and ^119^Sn NMR of complexes were recorded in deuterated CDCl_3_ on a Bruker Avance™ 400 MHz Spectrometer (Bruker, Karlsruhe, Germany; ^1^H, 400.23 MHz; ^13^C, 100.23 MHz; ^119^Sn NMR, 149.25 MHz). UV/Vis determination of the lipophilicity and stability of the complexes was carried out using a Shimadzu double-beam spectrophotometer (Shimadzu Deutschland GmbH, Duisburg, Germany) with a wavelength range of 200–500 nm at ambient temperature. The UV/Vis spectra were recorded at intervals of 24, 48, and 72 h.

#### 2.1.1. Synthesis of the Ligand Precursors (**HL1** and **HL2**)

**Scheme 1:**
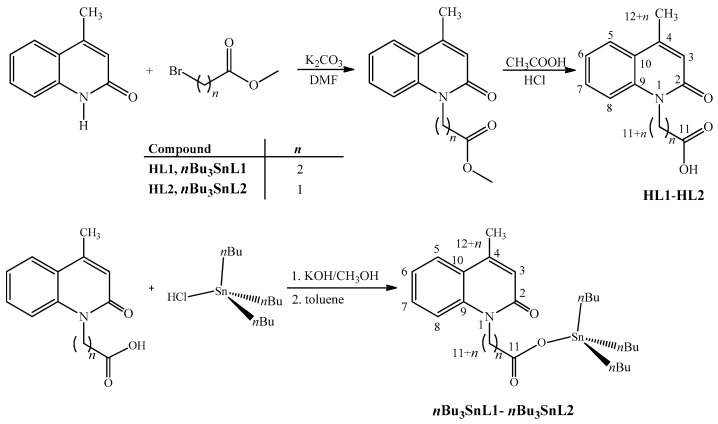
Synthesis of the ligand precursors, **HL1** and **HL2** [39], and tributyltin(IV) compounds, ***n*Bu_3_SnL1** and ***n*Bu_3_SnL2**. The numbering of atoms was used for NMR assignation and matched the one applied in the X-ray study of ***n*Bu_3_SnL2**.

Ligand precursors, 3-(4-methyl-2-oxoquinolin-1(2H)-yl)propanoic acid (**HL1**) and 2-(4-methyl-2-oxoquinolin-1(2H)-yl)acetic acid (**HL2**) were afforded by a two-step synthesis previously described in the literature [39]. In the first step, 2-quinolone (0.01 mol), K_2_CO_3_ (0.12 mol, Sigma-Aldrich Taufkirchen, Germany) and the corresponding halogen esters (0.011 mol) were added to 60 mL of *N,N*-dimethylformamide (Carl Roth, Karlsruhe, Germany). The reactants were mixed for 24 h at ambient temperature, then the resulting solution was poured into 180 mL of water, obtaining the white precipitate. The precipitates were extracted with ethyl acetate and the organic layer was washed with 5% NaOH solution. Ethyl acetate was removed by distillation, and the intermediate products were recrystallized from ethanol. A further step was the hydrolysis of the quinolone esters (0.01 mol) in glacial CH_3_COOH (40 mL, Carl Roth, Karlsruhe, Germany), with the addition of 10 mL of concentrated HCl (Carl Roth, Karlsruhe, Germany). The reaction mixture was refluxed for 2 h. The solvents were removed by vacuum distillation, and the procedure was repeated. Finally, the crude products were obtained, washed with water, and recrystallized with ethanol (Carl Roth, Karlsruhe, Germany) to yield pure crystals. The general synthesis pathway is illustrated in Scheme 1.

#### 2.1.2. Synthesis and Characterization of Tributyltin(IV) Compounds

Tributyltin(IV) complexes 3-(4-methyl-2-oxoquinolin-1(2H)-yl)propanoato)tributyltin(IV) (***n*Bu_3_SnL1**) and (2-(4-methyl-2-oxoquinolin-1(2H)-yl)ethanoato)tributyltin(IV) (***n*Bu_3_SnL2**) were synthesized according to the following procedure: to a suspension of ligand precursors, 0.5 mmol of **HL1** (115.62 mg), **HL2** (108.61 mg) in 5 mL of methanol (Carl Roth, Karlsruhe, Germany) was added a solid KOH (0.5 mmol, Sigma-Aldrich Taufkirchen, Germany), and deprotonation was carried out for 2 h at room temperature to obtain a clear solution. The methanol was removed under reduced pressure to dryness. Subsequently, 0.5 mmol of ***n*Bu_3_SnCl** (162.7 mg, Sigma-Aldrich Taufkirchen, Germany) dissolved in 5 mL of toluene was added, and the reaction mixture was stirred for 12 h. The solution was filtered to eliminate KCl and, due to the volatile behavior of the compounds, the solutions were left until the solvent content had decreased to 1 mL. The compounds were precipitated with dry *n*-hexane (Carl Roth, Karlsruhe, Germany), and then dissolved in dry toluene for crystallization. Suitable crystals of the compound ***n*Bu_3_SnL2** were afforded after slow evaporation of toluene (Carl Roth, Karlsruhe, Germany). The structure of the tributyltin(IV) compounds was investigated via FT-IR and NMR (^1^H, ^13^C, ^119^Sn) spectroscopy, whereas the structure of ***n*Bu_3_SnL2** was confirmed by X-ray crystallography diffraction analysis. The general procedure for synthesizing the ligand precursors and complexes is presented in Scheme 1.

(3-(4-Methyl-2-oxoquinolin-1(2H)-yl)propanoato)tributyltin(IV), ***n*Bu_3_SnL1**: Colorless sticky solid; Yield: 52%; Anal. calcd. for C_25_H_39_NO_3_Sn: C, 57.71; H, 7.56; N, 2.69; Found C, 57.62; H, 7.51; N, 2.76; Selected FT-IR data (ATR) cm^−1^: 2953, 2921, 2856, 1653, 1582, 1561, 1498, 1453, 1420, 1389, 1374, 1329, 1313, 1097, 868, 752, 664, 595, 537, 494, 444; ^1^H NMR (CDCl_3_, ppm): *δ* 7.72 (1H, dd, *J* = 7.9, 1.5 Hz; H^5^); *δ* 7.57 (1H, ddd, *J* = 8.5, 7.1, 1.5 Hz; H^7^); *δ* 7.49 (1H, d, *J* = 8.5 Hz; H^8^); *δ* 7.26 (1H, t, *J* = 7.5 Hz; H^6^); *δ* 6.59 (1H, s, H^3^); *δ* 4.58 (2H, m, H^13^); *δ* 2.74 (2H, m, H^12^); *δ* 2.47 (3H, s, -CH_3_); *n*-Bu-skeleton: *δ* 1.63 (6H, p, *J* = 7.8 Hz; H^α^); *δ* 1.37 (6H, m, H^β^); *δ* 1.29 (6H, m, H^γ^); *δ* 0.93 (9H, t, *J* = 7.3 Hz; H^δ^); ^13^C NMR (CDCl_3_, ppm): δ 19.04 (C^14^); δ 32.84 (C^12^); *δ* 38.81 (C^13^); *δ* 114.34 (C^8^); *δ* 121.1 (C^3^); *δ* 121.68 (C^10^); *δ* 121.82 (C^6^); *δ* 125.4 (C^5^); *δ* 130.52 (C^7^); *δ* 138.80 (C^9^); *δ* 146.48 (C^4^); *δ* 161.71 (C^2^); *δ* 176.30 (C^11^); *n*-Bu-skeleton: *δ* 13.66 (C^δ^); *δ* 16.51 (C^α^, ^1^*J*(^119^Sn–^13^C) = 358/343 Hz); *δ* 27.04 (C^γ^, ^2^*J*(^119^Sn–^13^C) = 64.64 Hz); *δ* 27.83 (C^β^, ^3^*J*(^119^Sn–^13^C) = 20.2 Hz); ^119^Sn NMR (CDCl_3_, ppm): 114.88.

(2-(4-Methyl-2-oxoquinolin-1(2H)-yl)ethanoato)tributyltin(IV), ***n*Bu_3_SnL2**: Colorless crystals; Yield: 57%; M.p.: 123–125 °C. Anal. calcd. for C_24_H_37_NO_3_Sn: C, 56.94; H, 7.37; N, 2.77; Found: C, 56.46; H, 7.49; N, 2.88; Selected FT-IR data (ATR) cm^−1^: 2955, 2919, 2852, 1634, 1569, 1503, 1455, 1437, 1396, 1373, 1341, 1305, 1078, 1026, 850, 754, 666, 608, 574, 516, 457; ^1^H NMR (CDCl_3_, ppm): *δ* 7.72 (1H, d, *J* = 8.0 Hz; H^5^); *δ* 7.51 (1H, t, *J* = 7.9 Hz; H^7^); *δ* 7.26 (1H, dd, *J* = 14.4, 6.8 Hz; H^8^); *δ* 7.16 (1H, d, *J* = 8.5 Hz; H^6^); *δ* 6.63 (1H, s, H^3^); *δ* 5.10 (2H, s, H^12^); *δ* 2.48 (3H, s, -CH_3_); *n*-Bu-skeleton: *δ* 1.56 (6H, p, *J* = 7.6 Hz; H^α^); *δ* 1.28 (12H, m, H^β^, H^γ^); *δ* 0.89 (9H, t, *J* = 7.3 Hz; H^δ^); ^13^C NMR (CDCl_3_, ppm): δ 19.09 (C^13^); δ 44.06 (C^12^); *δ* 114.24 (C^8^); *δ* 120.78 (C^3^); *δ* 121.50 (C^10^); *δ* 121.97 (C^6^); *δ* 125.37 (C^5^); *δ* 130.31 (C^7^); *δ* 139.28 (C^9^); *δ* 147.03 (C^4^); *δ* 161.65 (C^2^); *δ* 172.84 (C^11^); *n*-Bu-skeleton: *δ* 13.62 (C^δ^); *δ* 16.70 (C^α^, ^1^*J*(^119^Sn–^13^C) = 354/338 Hz); *δ* 26.95 (C^γ^, ^2^*J*(^119^Sn–^13^C) = 64.64 Hz); *δ* 27.68 (C^β^, ^3^*J*(^119^Sn–^13^C) = 21.21 Hz); ^119^Sn NMR (CDCl_3_, ppm): 129.51.

### 2.2. Crystal Structure Determination and Refinement

Data collection of crystals made available of ***n*Bu_3_SnL2** were hampered by the fact that crystals mounted at 100 K ruptured immediately leaving a microcrystalline material only. Most likely this is due to a low temperature phase transition. Rupturing was not observed if mounted at 200 K, thus sufficiently good intensity data of the crystals could be collected using a Rigaku Oxford Gemini S diffractometer (Rigaku, Tokyo, Japan). Selected crystallographic and structural refinement data of ***n*Bu_3_SnL2** can be found in Table S1. Despite the measurement temperature of 200 K, crystals turned out to be twinned. The finally selected crystals consisted of two domains, which contributed 55% and 45% to all data. The structure was solved by Direct Methods implemented within the WinGX software platform [42]. The model was refined with SHELXL-2013 (Version 2014.1) by full-matrix least square procedures on *F*^2^ using [43]. The positions of carbon bonded hydrogens were calculated at idealized calculated positions. Two of the *n*-butyl groups were refined disordered to split occupancies of 0.58/0.42 (C14–C18) and 0.38/0.62 (C22–C25) (Figure S1). CCDC 2383596 contains supplementary crystallographic data for ***n*Bu_3_SnL2**. These data are available free of charge from The Cambridge Crystallographic Data Centre via www.ccdc.cam.ac.uk.

### 2.3. Structure Optimization and Spectral Characterization

The structures of obtained compounds were optimized in the Gaussian 09 Program Package [44] employing the B3LYP-D3BJ [45,46] functional along with the 6-311++G(d,p) [47] basis set for H, C, O, and N atoms, and def2-TZV basis set for tin [48]. The optimizations were carried out without any geometric constraints for multiple starting structures, and the minima on potential energy surface was proven by the number of imaginary frequencies equal to zero. The most stable conformation was later used for the theoretical analysis of the compounds. The vibrations were visualized in the GaussView program [49]. The solvent effect was included through the CPCM model [50]. The NMR spectra of compounds have been projected by the GAIO method [51]. The ^1^H and ^13^C chemical shifts were referenced to tetramethylsilane (TMS), as in the experimental section, optimized at the same level of theory. The stabilization interactions were investigated within the Quantum Theory of Atoms in Molecules (QTAIM) approach, as proposed by Bader [52,53]. The AIMAll program package was employed for the QTAIM analysis [54].

### 2.4. Lipophilicity and Stability

To determine the lipophilicity of the compounds ***n*Bu_3_SnL1** and ***n*Bu_3_SnL2**, a flask-shaking method was applied for calculation of the partition coefficient log*P* [55]. This method implies the dissolving of compounds in DMSO and mixing with water/*n*-octanol system. The resulting mixture was exposed to vigorous vortexing for an hour at room temperature and left to stand for a further 24 h to allow the two phases to separate. The absorbance values were measured using calibration graphic and used to calculate the concentrations of tributyltin(IV) compounds in the *n*-octanol (*c*_o_) and water (*c*_w_) phases. These concentrations, applied in Equation (1), were used for calculation of log*P* value.

log*P* = log(*c*_o_*/c*_w_), (1)

### 2.5. In Vitro Studies

#### 2.5.1. Reagents and Cells

The reagents and cells were sourced from various manufacturers. RPMI-1640 culture medium and fetal bovine serum (FBS) were purchased from Capricorn Scientific GmbH (Ebsdorfergrund, Hessen, Germany). Paraformaldehyde (PFA) was obtained by Serva (Heidelberg, Germany). The penicillin–streptomycin solution was bought from Biological Industries (Cromwell, CT, USA), while 3(4,5-dimethylthiazol-2-yl)-2,5-diphenyltetrazolium bromide (MTT) was obtained from AppliChem (St. Louis, MO, USA). Dihydrorhodamine 123 (DHR 123) was procured from Thermo Fisher Scientific (Waltham, MA, USA). Matrigel^®^ was bought from BD Bioscience (Bedford, MA, USA). Crystal violet (CV), Ethylenediaminetetraacetic acid (EDTA), phosphate-buffered saline (PBS), Triton X-100, dimethyl sulfoxide (DMSO), Fluoromount-G, propidium iodide (PI), carboxyfluorescein diacetate succinimidyl ester (CFSE), and RNase were bought from Sigma (St. Louis, MO, USA). Annexin V-FITC (AnnV) was supplied by BD (Pharmingen, San Diego, CA, USA). Tumor cell lines (MCF-7 (human breast adenocarcinoma), HCT116 (human colorectal carcinoma), A375 (human melanoma), 4T1 (mouse breast carcinoma), CT26 (mouse colon carcinoma), B16 (mouse melanoma)) were obtained from the American Type Culture Collection (ATCC, Manassas, VI, USA).

All cell lines were cultured in HEPES-buffered RPMI-1640 medium supplemented with 10% heat-inactivated FBS, 100 units/mL penicillin, and 100 μg/mL streptomycin. The cells were cultured in a conventional environment at 37 °C, in a humidified atmosphere with 5% CO_2_. In order to assess the cell, viability cells were seeded in 96-well plates at the following densities: B16 (3 × 10^3^ cells/well), 4T1 (4 × 10^3^ cells/well), CT26, HCT116 and A375 (5 × 10^3^ cells/well), and MCF-7 (8 × 10^3^ cells/well). In 6-well plates, flow cytometric analyses were conducted with A375 cells, and the cell density was adjusted to 1.5 × 10^5^ cells per well.

#### 2.5.2. Determination of Cell Viability (MTT and CV Assays)

The cell lines were cultured overnight and exposed to the compounds ***n*Bu_3_SnL1** and ***n*Bu_3_SnL2**. After a 72 h incubation period, cell viability was estimated by MTT and CV tests exactly as described [41]. The results were normalized to the control, which was assigned a value of 100%.

#### 2.5.3. Annexin V/Propidium Iodide (PI)

To identify apoptosis, A375 cells were exposed to IC_50_ concentrations of compounds ***n*Bu_3_SnL1** and ***n*Bu_3_SnL2** for 48 and 72 h. After the specified incubation period, the cells were rinsed with PBS and stained with Annexin V and propidium iodide, each at a final concentration of 15 μg/mL, for 15 min at room temperature, protected from light. The cells were then resuspended in Annexin V-binding buffer (ABB) and analyzed by flow cytometry using the CytoFLEX Flow Cytometer (Beckman Coulter, Life Sciences, Indianapolis, IN, USA).

#### 2.5.4. PI Staining on Chamber Slides

To evaluate the morphological indicators of apoptosis, A375 cells were cultured overnight in 4-chamber slides at a density of 7 × 10^4^ cells and exposed to a compound ***n*Bu_3_SnL1** and ***n*Bu_3_SnL2** at their respective IC_50_ doses for 72 h. Following the incubation period, the cells were rinsed with PBS, fixed with 4% PFA for 15 min at ambient temperature, and then washed several times with PBS. Subsequently, the cells were stained with a solution containing PI (50 μg/mL), 0.1% Triton X-100, 0.1 mM EDTA (pH 8.0), and RNase (85 μg/mL) in PBS for 1.5 min. Lastly, the slides were mounted with fluorescent mounting medium, and the analysis was conducted using a Zeiss Axio Observer Z1 inverted fluorescence microscope (Carl Zeiss AG, Oberkochen, Germany) at a magnification of 400×.

#### 2.5.5. Cell Adhesion Assay In Vitro

A375 cells were cultivated in 25 cm^2^ flasks and exposed to a subtoxic dose (IC_25_) of compounds ***n*Bu_3_SnL1** and ***n*Bu_3_SnL2** for 72 h. After 72 h, the cells were trypsinized, collected, and kept at RT for 30 min in order to reconstruct the cell membranes. Matrigel^®^ (50 μL/well) was used for coating the 96-well plates (20 μg/mL) and plates were left overnight at 4 °C in order for Matrigel^®^ to polymerize. Wells were washed several times with PBS prior to seeding the cells at a density of 3 × 10^4^ cells/well and cells were kept at 37 °C to adhere for 1 h. PBS was used to wash unattached cells, and the number of remaining adherent cells was determined using CV test. The absorbance was measured at 570 nm/670 nm and relative cell adhesion was quantified.

### 2.6. Spectrofluorometric BSA Binding Affinity

The experimental BSA binding affinity was investigated on a Cary Eclipse MY2048CH03 instrument through successive addition of obtained compounds. The solutions of BSA with and without added compounds were excited by 295 nm, which is specific for the tryptophan residues in the BSA structure. The emission spectra were followed in the range 310–500 nm. Other parameters include the scan rate of 600 nm min^−1^ and slits of 5 nm. The concentration of protein was 5 × 10^−6^ M in the phosphate-buffered saline (pH = 7.4) [41], while the concentration of organotin compounds ranged between 1 and 10 × 10^−6^ M. Upon the addition of complexes, the change in fluorescence emission intensity with concentration followed a double-log Stern–Volmer quenching equation:

(2) $$\log\left(\frac{I_{0}-I}{I}\right)=\log K_{b}+n\log\left[Q\right]$$

In the previous equation, *I*_0_ and *I* represent the fluorescence intensities of BSA in the absence and presence of quenchers, respectively. *K_b_* refers to the binding constant, *n* represents the number of binding positions, and [*Q*] is the concentration of tin compounds. The thermodynamic parameters of binding, namely change in enthalpy (Δ*H_b_*) and entropy (Δ*S_b_*) were obtained through the following equation for measurements at three temperatures (27, 32, and 37 °C):

(3) $$\ln K_{b}=-\frac{∆H_{b}}{RT}+\frac{∆S_{b}}{R}$$

### 2.7. Molecular Docking

Molecular-level investigations of the binding interactions between obtained complexes and BSA were carried out using molecular docking simulations with AutoDock 4.2.6 [56], with the assistance of the AMDock program (version 1.5.2) [57]. The structures of organotin(IV) compounds were optimized at the B3LYP-D3BJ/6-311++G(d,p)(H,C,N,O)/def2-TZVP(Sn) basis sets. The structure of BSA (PDB ID: 4OR0 [58]) was obtained from PDB. Molecular preparation, docking setup, and the visualization and analysis of docking results were performed using BIOVIA (2019) Discovery Studio [59].

## 3. Results and Discussion

### 3.1. Vibrational Spectroscopy

Characteristic infrared frequencies were utilized to determine the coordination behavior of the carboxylic group of the newly synthesized compounds (Figure S2). The disappearance of the prominent ν(COOH) peak in the 1715–1730 cm^−1^ region in the spectra of the free ligand precursors, and the appearance of asymmetric [ν_asym_(COO^−^)] and symmetric [ν_sym_ (COO^−^)] stretching bands, imply the formation of tributyltin(IV) complexes and the coordination of the carboxylic group to the Sn-atom [39]. In the case of the ***n*Bu_3_SnL1**, two asymmetric stretching bands (1582 and 1561 cm^−1^) and two symmetric stretching bands (1389 and 1374cm^−1^) are observed. For the compound ***n*Bu_3_SnL2**, only one pair of asymmetric and symmetric (1569 and 1341 cm^−1^) vibrations were noticed. The value of Δν (Δν = ν_asym_ − ν_sym_) for compound ***n*Bu_3_SnL1** is under 200 cm^−1^, suggesting the bridging bidentate mode of the coordination of the carboxylic group [60]. The magnitude of the Δν separation for complex ***n*Bu_3_SnL2** is 227 cm^−1^, which indicates that carboxylate moiety coordinates in a monodentate manner. Furthermore, the presence of the tributyl moiety is identified among stretching bands in the 2955–2852 cm^−1^ region, while the bands at 595 and 574 cm^−1^ are assigned to ν(Sn–C). Stretching vibrations at 444 (***n*Bu_3_SnL1**) and 456 cm^−1^ (***n*Bu_3_SnL2**) are assigned to ν(Sn–O) [21,61].

### 3.2. NMR Spectroscopy

The ^1^H NMR resonances for the aromatic protons of the quinolone backbone are observed between 6.59 and 7.72 ppm. The methylene protons in α-position relative to the carboxylic group show chemical shifts to higher value by 0.09–0.19 ppm when compared to deprotonated ligands [39]. The resonance of methylene and methyl protons attributed to the tributyltin(IV) moiety are assigned by comparison with similar tributyltin(IV) compounds [61]. In tributyltin(IV) complex ***n*Bu_3_SnL1** (Figure S3), α and β protons from tributyltin(IV) moiety show two clearly visible pentets at 1.63 and 1.37, γ protons are observed as multiplet at 1.29 ppm, whereas terminal methyl groups give sharp triplet at 0.93 ppm. Similarly, in the case of ***n*Bu_3_SnL2** (Figure S4), α protons show pentet at 1.55 ppm, β and γ protons are associated in multiplet in the range from 1.35–1.22 ppm, and terminal methyl groups give sharp triplet at 0.89 ppm.

The ^13^C NMR signals of the methyl and methylene carbon atoms from tributyltin moiety are attributed according to the coupling constants ^1^*J*(^119^Sn–^13^C), ^2^*J*(^119^Sn–^13^C) and ^3^*J*(^119^Sn–^13^C), and are in agreement with similar compounds [60]. The resonances of the carboxylic carbon in complexes ***n*Bu_3_SnL1** and ***n*Bu_3_SnL2** are shifted upper for 0.46 and 0.88 ppm, compared to deprotonated ligand precursors. Coupling constants ^1^*J*(^119^Sn–^13^C) are noteworthy factors for the structural elucidation of organotin(IV) complexes. The values of ^1^*J*(^119^Sn–^13^C) for tetracoordinated tetrahedral tributyltin(IV) complexes in non-coordinating solvents are reported to be lower than 400 Hz [62]. For compounds ***n*Bu_3_SnL1** and ***n*Bu_3_SnL2**, coupling constants ^1^*J*(^119^Sn–^13^C) have values of 358/343 and 354/338 Hz, supporting the tetrahedral environment around tin atom [22].

The ^119^Sn NMR spectral data further confirms the tetrahedral arrangement around the Sn atom in non-coordinating solvents. The ^119^Sn NMR resonances for ***n*Bu_3_SnL1** and ***n*Bu_3_SnL2** display sharp signals at 114.88 and 129.51 ppm, and are in agreement with those similar tetracoordinated tributyltin(IV) carboxylate complexes [63].

### 3.3. Crystallographic Structure

**Figure 1:**
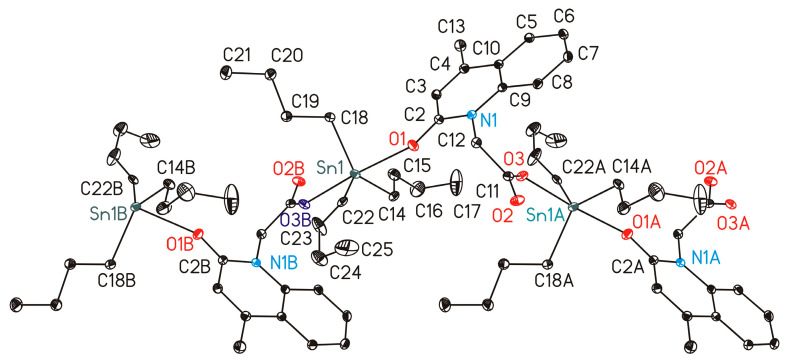
ORTEP diagram (25% probability ellipsoids) of a selected part of the chain formed by ***n*Bu_3_SnL2** in the solid state. All hydrogen atoms are omitted for clarity and, of disordered atoms, only one atomic position is shown. Symmetry codes: “A” = ½ − *x*, *y* + ½, ½ − *z*; “B” = ½ − *x*, −½ + y, ½ − *z*.

The perspective view of the molecular structure of the compound ***n*Bu_3_SnL2** is presented in Figure 1, and the corresponding bond lengths and angles are listed in Table S2. The tributyltin(IV) compound ***n*Bu_3_SnL2** crystallizes in the monoclinic system with the *P2_1_/n* space group, resulting in the formulation of a one-dimensional chain. The geometry around the Sn atom is trigonal bipyramidal, with three tributyl groups occupying the equatorial positions. The axial positions are occupied with one oxygen atom derived from the carboxylic group, and another oxygen atom from the keto group bonded for quinolone moiety. According to the literature, the degree of distortion for five-coordinated setups can be quantitatively characterized by the parameter *τ*, where the value of *τ* = 1 corresponds to a perfect trigonal bipyramidal geometry, and the value of *τ* = 0 corresponds to a square-pyramidal geometry [13,16]. The calculated value of *τ* for compound ***n*Bu_3_SnL2** is 0.74, indicating a highly distorted trigonal bipyramidal arrangement around the Sn atom, which is probably due to the bulky butyl groups bonded to the Sn center [63]. The total of the equatorial C–Sn–C angles is 358.8°, and the axial angle is 170.6°.

### 3.4. Theoretical Analysis of Structures and Prediction of the NMR and IR Spectra

**Figure 2:**
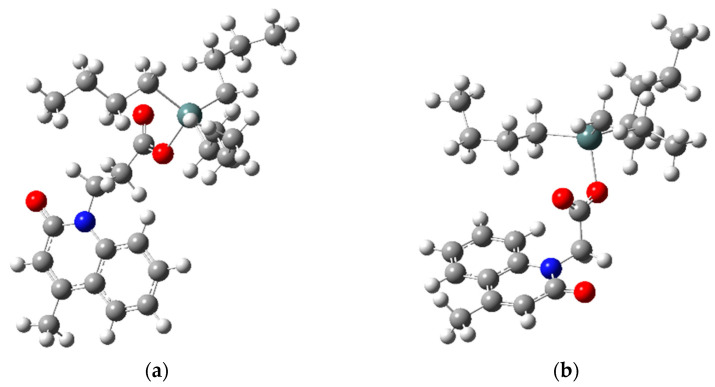
Optimized structures of organotin compounds at B3LYP-D3BJ/6-311++G(d,p)(H,C,N,O)/def2-TZVP(Sn) level of theory: (**a**) ***n*Bu_3_SnL1**, (**b**) ***n*Bu_3_SnL2** (Hydrogen—white, carbon—gray, nitrogen—blue, oxygen—red, tin—dark green).

The crystallographic structure of ***n*Bu_3_SnL2** was taken as the initial structure for optimization at the level of theory mentioned in the Methodology section. Theoretical and experimental structural parameters were compared to verify the applicability of the chosen level of theory, as these structures were further used for the molecular docking simulations. These data sets were examined by determining the correlation coefficient (R) and the mean absolute error (MAE), as described in the previous contribution on similar compounds [40]. The experimental and theoretical values are listed in Tables S2 and S3, and the structures of ***n*Bu_3_SnL1** and ***n*Bu_3_SnL2** are shown in Figure 2.

The correlation coefficients for comparing experimental and theoretical bond lengths and angles of ***n*Bu_3_SnL2** are 0.99 and 0.86, respectively. When closer inspection of the bond lengths was performed, it was concluded that the MAE value was 0.03 Å. Due to the structural rigidity of the 4-methyl-2-oxoquinolinyl moiety, the differences between the two data sets were low. The Sn–O bond length in crystallographic structure was 2.15 and theoretical 2.11 Å. The bonds between carbon atoms and Sn ranged between 2.05 and 2.12 Å (experimental) and around 2.16 Å (theoretical structure). This equilibration of bonds in an optimized structure results from the system relaxation and adaptation of the tetrahedral geometry. Additional interactions between Sn and carbonyl oxygen in the crystallographic structure greatly influence the overall geometry. This is especially visible when optimized and crystallographic bond angles are compared, leading to a lower correlation coefficient (0.86) and higher MAE value (2.4°). The bond angle denoted as C^11^–O–Sn was 128.5° in the experimental and 116.7° in the theoretical structure. Significant differences were observed in the bond angles surrounding the Sn atom, including carbon atoms of butyl chains and oxygen atoms of the carboxyl group, on an average of 9°. Upon optimization, these angles were almost equal. As already discussed, the additional interaction distorts the geometry of the compound in the solid state. In this contribution, these interactions were not considered during optimization. In addition, interactions with other units within crystal structure packaging can influence the rotation of the butyl groups. The presented results indicate that the theoretical structure describes the experimental one well and can be used in subsequent analysis.

It is beneficial to discuss the structural differences between ***n*Bu_3_SnL1** and ***n*Bu_3_SnL2**_,_ as the crystallographic structure of the first compound was not obtained. The MAE values, when bond lengths and angles of these two compounds were compared, are 0.002 Å and 0.6°, which proves that the additional CH_2_ group does not influence the overall geometry significantly. The most notable differences were found for the angles around the Sn atom, between 1.6 and 4.0°, which can be attributed to the flexibility of the aliphatic chain that connects the 4-methyl-2-oxoquinolinyl moiety and the carboxyl group. The loss of extended delocalization within this part of the molecule induces the possibility of a relative change in position between these two groups.

The interactions between Sn and the surrounding carbon and oxygen atoms were further examined using the QTAIM approach. The bond critical points (BCPs) of interest were characterized by the electron density (ρ(r)), Laplacian (*∇*^2^ρ(r)), Lagrangian kinetic electron density (*G*(r)), potential electron density (*V*(r)), density of total electron energy (*H*(r) = *G*(r) + *V*(r)), and interatomic bond energy (E_bond_), as presented in [40]. These values are given in Table S3, and the graphical representation of the BCPs of interest is in Figure S5.

Two types of stabilization interactions between ligands and central atoms were found in the structures. The first type includes Sn–O and Sn–C interactions, with positive electron density values (0.085–0.106 a.u.) and positive Laplacian values (0.074–0.326 a.u.). The electron densities are lower, while Laplacian values are higher in the case of Sn–O when compared to Sn–C, proving the relative strength of these interactions. The presented range of values indicates that these interactions can be considered closed-shell interactions (covalent bonds) [40]. The values of the ratio −(*G*(r)/*V*(r)) for these bonds, between 0.59 and 0.87, and interatomic bond energy higher than −160.0 kJ mol^−1^, are additional proof of the covalent character. The interaction energy has a higher absolute value for Sn–O, although it should be noted that for ***n*Bu_3_SnL2**, all four interactions around the Sn atom have similar bond energies, which is a consequence of the spatial distribution of the atoms and ligand structure. When bond energies of ***n*Bu_3_SnL1** are examined, the larger difference can be found due to the change in ligand structure, although bond energies of Sn–C are the same.

Additional interactions are formed between the carbonyl oxygen and hydrogen atoms of butyl chains and the benzene ring of quinoline moiety and hydrogen atoms in the structure of ***n*Bu_3_SnL2**. These interactions are characterized by the much lower electron density and Laplacian values. Positive *H*(r) values and positive values of the ratio –(*G*(r)/*V*(r)) classify these interactions as closed-shell van der Waal interactions [40]. The bond energies of these interactions are between −3.65 and −6.9 kJ mol^−1^. Interactions between carbonyl oxygen and hydrogen atoms are stronger, as expected. These interactions are formed because of the proximity of the ligand groups. The elongated structure of ***n*Bu_3_SnL1** limits the number of interactions (Figure S5) to only two, one including the carbonyl oxygen (−5.3 kJ mol^−1^), the other through the benzene ring (−2.0 kJ mol^−1^). Although weak, these interactions are important for the compounds’ overall structural stability and conformation preferences in the gas phase. It can be expected that interactions with solvent molecules will be much more dominant in the solution.

**Figure 3:**
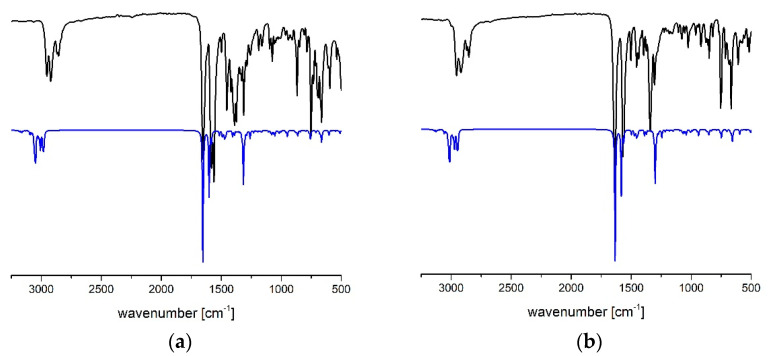
Experimental (black) and theoretical IR spectra (blue) of investigated compounds: (**a**) ***n*Bu_3_SnL1**, (**b**) ***n*Bu_3_SnL2** (at B3LYP-D3BJ/6-311++G(d,p)(H,C,N,O)/def2-TZVP(Sn) level of theory).

The vibrational spectra of ***n*Bu_3_SnL1** and ***n*Bu_3_SnL2** were predicted starting from the optimized structures to assign the most prominent bands in the experimental spectra (Figure 3). The vibrations of optimized structures were visualized in the GaussView program. The theoretical values were overestimated since theoretical spectra were obtained for isolated compounds in a vacuum, whereas the experimental ones were obtained for solid samples. The correction factors were calculated by comparing the wavenumbers of the most intense peaks.

The region of the spectrum from 3500 to 2000 cm^−1^ covers the stretching C–H vibrations, and the position of peaks depends on the hybridization of carbon atoms. Due to the overall symmetry of obtained compounds, these peaks have low intensity. The bands at 3049 and 3012 cm^−1^ in the theoretical spectrum are attributed to the C–H stretching vibrations of quinoline moiety. The methyl group and C–H vibrations of the aliphatic chain are located above 3000 cm^−1^ due to the presence of unsaturated bonds and electronegative oxygen atoms. The butyl group C–H stretching vibrations are present in the experimental and theoretical spectra below 3000 cm^−1^.

The most intense peaks in the spectra of both compounds are present in the second region, between 1800 and 1000 cm^−1^, as previously discussed for similar compounds [40,41]. The peaks originating from C=O stretching vibrations are found in the following positions of the experimental/theoretical spectra of ***n*Bu_3_SnL1**: 1655/1653 and 1601/(1582 and 1561) cm^−1^. The higher position is assigned to the carbonyl group that is part of the quinoline moiety. At the same time, the latter one belongs to the carboxyl group vibrations, as the frequency decreases due to the deprotonation of this group. These bands are positioned at 1633/1634 and 1583/1569 cm^−1^ in the spectra of ***n*Bu_3_SnL2**. Lower frequencies in the second compound’s spectra result from the elongated conjugation within the aliphatic chain. The coordination to the Sn atom induces a bathochromic shift in the position of C=O stretching bands located at 1389/1374 and 1341 cm^−1^ in the experimental spectra of ***n*Bu_3_SnL1** and ***n*Bu_3_SnL2**. The same bands are found at 1315 and 1298 cm^−1^ in the theoretical spectra. The difference between experimental and theoretical values can be attributed to the strong influence of crystal packaging and hindered vibrations due to interactions with surrounding units.The region below 1000 cm^−1^ usually contains the out-of-plane and torsional vibrations of compounds characteristic of the backbone structure. As previously mentioned, the Sn–C vibrations in the experimental spectra of ***n*Bu_3_SnL1** and ***n*Bu_3_SnL2** have characteristic wavenumbers at 595 and 574 cm^−1^. Due to the local symmetry of carbon atoms around Sn, the calculated wavenumbers of symmetric vibrations of Sn–C bonds are at 571/578 cm^−1^, while asymmetric vibrations are at 602/595 cm^−1^ in the spectra of both compounds. The Sn–C stretching vibration wavenumbers are also well reproduced at 498 cm^−1^ in both spectra.

The experimental and theoretical ^1^H and ^13^C NMR chemical shifts were also compared as additional proof that the optimized structures reflect the experimental characteristics well. The experimental spectra were recorded in CDCl_3_, while the theoretical structures were reoptimized in the same solvent, and the chemical shift values were calculated relative to TMS. The same set of parameters was used to compare these two data sets. The obtained chemical shifts were consistently overestimated, and the correction factor was determined based on the dependency of theoretical values on experimental values. These correction factors were 0.99 and 0.93 for ^1^H and ^13^C NMR chemical shifts, respectively. The differences between these data sets resulted from the optimization of isolated molecules in a vacuum, which differs from the solution with solvent–solute interactions present, together with the effect of local vibrations and rotations of specific groups. The experimental and scaled values of chemical shifts are given in Table 1 and Table S4.

The structures of newly obtained organotin(IV) compounds are characterized by two distinct groups of protons in the ^1^H NMR spectra. The first group includes the alkyl proton as part of the methyl and butyl groups. The position of methyl protons is at 2.47 and 2.48 ppm in the experimental and 2.42 and 2.45 ppm in the theoretical spectra of ***n*Bu_3_SnL1** and ***n*Bu_3_SnL2**, respectively. The position of the butyl group protons depends on the proximity of tin(IV), and it decreases with the distance from 1.63 to 0.93 ppm in the experimental and from 1.11 to 0.84 ppm in the theoretical spectra of the first compound. A similar effect was found in the spectra of the second compound. Hydrogen atoms attached to the aliphatic chain in positions 12 and 13 of ***n*Bu_3_SnL1** and in position 12 of ***n*Bu_3_SnL2** have higher values due to the proximity of the carboxyl group. The quinoline moiety protons have higher values characteristic for the protons of aromatic groups, between 6.59 and 7.72 ppm in the experimental and between 6.49 and 8.12 ppm in the theoretical spectra of ***n*Bu_3_SnL1** (Table 1). The values of the chemical shifts of protons in positions are partially influenced by the presence of a carbonyl oxygen atom. In the case of ***n*Bu_3_SnL2**, a similar range of values was obtained. Nevertheless, the theoretical values describe the experimental ones well, with a correlation coefficient higher than 0.98 and an MAE value between 0.28 and 0.33 ppm.

The ^13^C NMR chemical shifts are reproduced even better, with a correlation coefficient equal to 1.00 and MAE values of 1.92 (***n*Bu_3_SnL1**) and 2.28 ppm (***n*Bu_3_SnL2**). The lowest values of chemical shift values were obtained for the carbon atoms of the butyl and methyl groups, although the actual values depended on the proximity of tin and quinoline moiety. The signals of the butyl group carbon atoms were found between 13.66 and 27.83 (experimental) and between 15.64 and 31.35 ppm (theoretical) in the spectra of ***n*Bu_3_SnL1**. These values were not influenced by the elongation of the aliphatic chain of ligand molecules. The methyl group carbon atom is characterized by the signals at 19.04 and 19.09 ppm in the experimental spectra of both compounds. These values are higher due to the presence of quinoline moiety. The carbon atoms in position 3 have chemical shift values of 38.81 and 43.27 ppm in the experimental and theoretical spectra of ***n*Bu_3_SnL1**. Other carbon atoms of quinoline moiety have much higher values of chemical shifts, between 114.34 and 146.48 ppm in the spectrum of ***n*Bu_3_SnL1**. Similar values were found in the spectra of ***n*Bu_3_SnL2**. The highest values were obtained for the carbon atoms in positions C^4^, C^2^, and C^11^, due to the effect of electronegative oxygen atoms. The chemical shift values of C^2^ are higher than those of C^4^, as the methyl group acts as an electron donor, as previously discussed for similar compounds [40,41]. The values of chemical shifts of C^11^ are 176.30 and 174.66 ppm in the spectra of ***n*Bu_3_SnL1** and 172.84 and 173.14 ppm in the spectra of ***n*Bu_3_SnL2**. The effect of coordination to Sn atoms is visible in chemical shifts of C^11^ and C^α^ atoms. These spectral analyses have proven that the optimized structures can be further used to investigate interactions with transport proteins.

### 3.5. Lipophilicity and Stability

Cytotoxicity of organotin(IV) compounds is correlated to lipophilicity, and the most lipophilic compounds are the most cytotoxic. In drug discovery structural activity relationship studies, lipophilicity is a commonly used parameter, since the structural modifications that change lipophilicity affect solubility, metabolic stability, absorption, bioavailability, and toxicity [64,65,66]. However, the application of high lipophilic compounds can cause hazard effects, due to increased permeability and toxicity [67]. According to the Comprehensive Medicinal Chemistry database, compounds that possess log*P* value in the range from –0.4 to 5.6 are qualified for potential medicinal use [68]. Tributyltin(IV) compounds ***n*Bu_3_SnL1** and ***n*Bu_3_SnL2** have log*P* values of 1.23 and 1.54, respectively, in perfect range, which qualify these compounds for further evaluation of in vitro/in vivo biological activity and potential clinical trials. Moreover, the molecular weight of ***n*Bu_3_SnL1** and ***n*Bu_3_SnL2** follows the Lipinski’s rule of five for orally used therapeutic agents [69].

The stability of the complexes ***n*Bu_3_SnL1** and ***n*Bu_3_SnL2** was examined by measuring UV/Vis in the water/DMSO solvent mixture, immediately after dissolution and at 24, 48, and 72 h intervals (Figure S6). The maximum absorbance for both compounds was shifted during the measurements (after 72 h), which may indicate the occurrence of hydrolysis of the tributyltin(IV) complexes.

### 3.6. Cytotoxic Potential of nBu_3_SnL1 and nBu_3_SnL2 In Vitro and Comparison to Related Organotin(IV) Compounds

To evaluate the effect of experimental compounds on cell viability, various concentrations of ***n*Bu_3_SnL1** and ***n*****Bu_3_SnL2** were applied to several cancer cell lines different in their origin and type (MCF-7, A375, HCT116, 4T1, B16, and CT26, see Figures S7 and S8). After 72 h of incubation, MTT and CV assays were applied. The data indicated a decrease in cell viability across all cell lines, with IC_50_ values observed within the nanomolar range (Table 2).

The novel compounds, ***n*Bu_3_SnL1** and ***n*Bu_3_SnL2**, demonstrated significantly higher activity against tumor cell lines compared to **HL1** and **HL2**, with enhancements ranging from 250 to 1000 times (CV assay). This increase in efficacy also surpasses **Me_3_SnL1** by tenfold (CV assay). The activity levels of ***n*Bu_3_SnL1** and ***n*Bu_3_SnL2** are comparable to those observed for diphenyltin(IV) compounds, **Ph_2_Sn(L1)**_2_ and **Ph_2_Sn(L2)_2_**, but lower than the corresponding triphenyltin(IV) compounds, **Ph_3_SnL1** and **Ph_3_SnL2**. In general, the activity hierarchy within this class of ligands in organotin(IV) compounds is as follows: **Me_3_SnL** < ***n*Bu_3_SnL** ≈ **Ph_2_Sn(L)_2_** < **Ph_3_SnL** (Table 2). Notably, ***n*Bu_3_SnL1** and ***n*Bu_3_SnL2** exhibited higher activity than cisplatin, ranging from 15 to 90 times (CV assay). Importantly, ***n*Bu_3_SnL1** and ***n*Bu_3_SnL2** showed no detrimental effects on the viability of primary peritoneal exudate cells (PEC, Figure S9), indicating high degree of selectivity towards malignant cells.

### 3.7. Mechanism of ***nBu_3_SnL1*** and ***nBu_3_SnL2***

To determine the presence of apoptotic/necrotic cell death in response to treatment, A375 cells were exposed to IC_50_ dose of ***n*Bu_3_SnL1** or ***n*Bu_3_SnL2** for 72 h, the same time interval chosen to assess cell viability.

**Figure 4:**
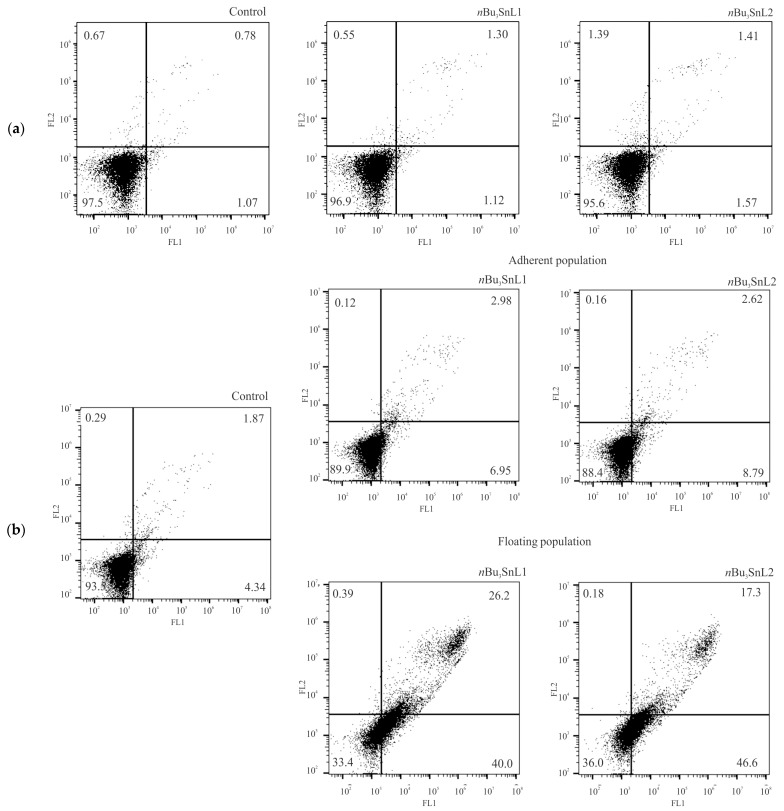
Compounds ***n*Bu_3_SnL1** and ***n*Bu_3_SnL2** induced anchorage independent apoptosis in A375 cultures. A375 cells were exposed to an IC_50_ dose of compounds ***n*Bu_3_SnL1** and ***n*Bu3SnL2**, and flow cytometry of Ann/PI-stained cells was performed after (**a**) 72 h treatment and (**b**) 48 h treatment.

**Figure 5:**
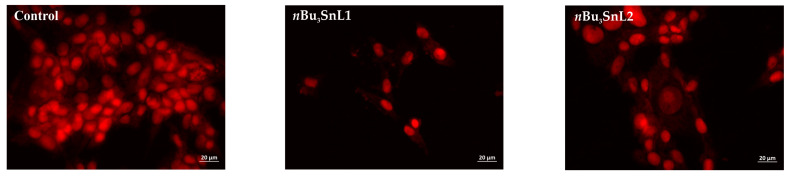
PI staining on chamber slides after 48 h treatment of A375 cells with compounds ***n*Bu_3_SnL1** and ***n*Bu_3_SnL2** (IC_50_ dose).

Cells were subsequently stained with Annexin V/propidium iodide, a sensitive method for the detection and distinction between early and late apoptotic/necrotic cells. Unexpectedly, due to powerful cytotoxicity determined by viability assay, no significant accumulation of apoptotic cells was detected upon flow cytometric analysis of Ann/PI double-stained cells at the indicated time point (Figure 4a). To exclude the possibility that the apoptotic cell population was lost during the staining procedure as a result of spontaneous cell degradation following a prolonged breakdown of cell permeability, the cultivation period was shortened for 24 h. In agreement with this assumption, the massive presence of cells with typical apoptotic phenotype, characterized by phosphatidylserine externalization in the early phase and diminished cell permeability in the late phase, classified as Ann^+^/PI^−^ and Ann^+^/PI^+^, respectively, was detected only in the floating cell fraction (Figure 4b). The corresponding adherent subpopulation was almost completely viable (Figure 4b). In line with this, PI staining of A375 cells cultivated on chamber slides after 48 h of treatment with ***n*Bu_3_SnL1**, or alternatively ***n*Bu_3_SnL2**, confirmed only the sporadic presence off typical apoptotic cells in the thin adherent population (Figure 5).

**Figure 6:**
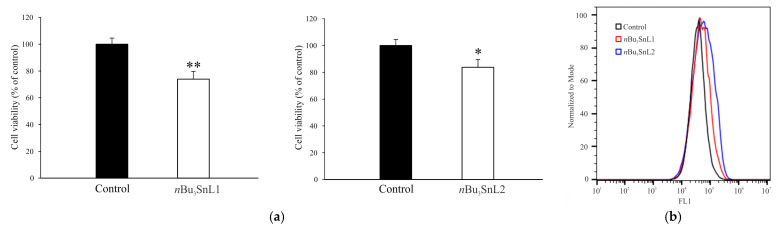
Compounds ***n*Bu_3_SnL1** and ***n*Bu_3_SnL2** affected the adhesion and ROS/RNS production. (**a**) A375 cell adhesion on Matrigel^®^ and (**b**) ROS/RNS production (* and ** are for *p* < 0.05, *p* < 0.01, respectively).

Although it is known that apoptotic cells begin to disintegrate in the late phase and consequently separate from the bottom of the plate, the presence of early apoptotic cells that still have the property of adhesion is expected, while the floating subpopulation mainly contains double positive cells. The massive cell detachment visible after 48 h of cultivation in the presence of selected compounds display apoptotic features typical of both early and late stages, indicating that cell detachment probably occurred prior to the execution of apoptotic cell death. This chronology is typical for the anchorage-independent type of apoptosis known as anoikis. To support this hypothesis, the influence of the tested compounds on cell adhesion properties was investigated and a cell adhesion assay was performed. To avoid falls positive results and cell detachment caused by drug toxicity, cells were exposed to an IC_25_ dose of each of the tested compounds. As can be seen in Figure 6a, the adhesion of A375 cells to the extracellular matrix was significantly impaired by treatment with both compounds, leading to the conclusion that the cells exposed to the experimental therapeutics lost their anchorage and consequently entered the apoptotic program. It is well reported that loss of cellular attachment resulted in ‘anoikis’, a form of apoptotic cell death, one of the essential mechanism for preventing detached cells surviving and abnormal growth in inappropriate places [70]. Results confirming abrogated adhesive potential of A375 cells are consistent with the hypothesis made on the basis of Ann/PI staining and data collected after flow cytometric analysis of floating population after 48-h long treatment with each of the compounds. Taken together, this suggests that selected agents induce anchorage independent apoptosis.

Further investigation of reactive oxygen species (ROS) and reactive nitrogen species (RNS) production in A375 cells revealed that both ***n*Bu_3_SnL1** and ***n*Bu_3_SnL2** moderately stimulated production of reactive molecules (Figure 6b). Since it is reported that ROS/RNS acts as secondary messengers of integrin signaling, changes in their production can affect cell adhesiveness and subsequent cell detachment, resulting in anchorage independent apoptosis [71].

The importance of efficient anoikis induction is connected with prevention of metastasis. While dissemination of cancer cells mainly relies on the anoikis resistance of cancer cells, a very promising approach in advanced cancer treatment is efficient induction of this type of cell death [35].

The novel compounds ***n*Bu_3_SnL1** and ***n*Bu_3_SnL2** demonstrated significant cytotoxic activity against A375 cells, exhibiting different mechanisms of action compared to previously studied compounds such as **Me_3_SnL1**, **Ph_3_SnL1** and **Ph_3_SnL2**, based on induction anchorage independent apoptosis. This study underscores the importance of the organic moiety R bonded to tin(IV) component in **R_3_SnL**, in addition to the ligand **L**, in determining the mechanism of cell death. For these specific organotin(IV) compounds bearing carboxylato *N*-functionalized 2-quinolones, the presence of Me groups in R_3_SnL influence on autophagy induction via lipid peroxidation. The *n*Bu moieties probably activate anoikis by increasing ROS/RNS levels and disrupting cell adhesion. Ph groups showed a different mechanism by promoting apoptosis and inhibiting ROS/RNS production in A375 cells. Unlike the presence of Me or Bu groups, which increase ROS/RNS, organotin(IV) compounds with Ph groups inhibited ROS/RNS production. This indicates that the R component of these compounds has a substantial impact on the mechanism of cell death, beyond the effects of the organic ligand alone.

Overall, ***n*Bu_3_SnL1** and ***n*Bu_3_SnL2** exhibit considerable potential as anticancer agents due to their high efficacy against tumor cell lines, selective action towards malignant cells, and their ability to induce anchorage independent apoptosis and reduce cell adhesion. These findings warrant further studies to elucidate the precise mechanisms underlying their cytotoxic activity and to evaluate their potential in in vivo models [72].

### 3.8. Experimental and Theoretical Protein Binding Affinity

**Figure 7:**
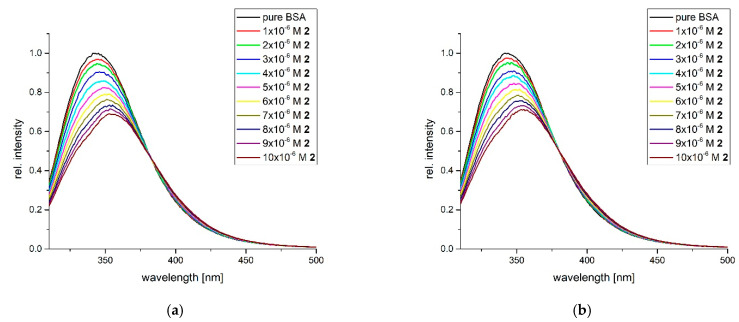

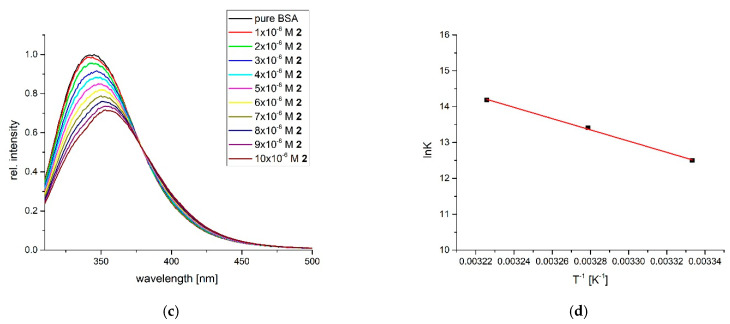
Fluorescence emission spectra of BSA for the titration with ***n*Bu_3_SnL2** at (**a**) 27°, (**b**) 32°, (**c**) 37°, and (**d**) Van ’t Hoff plot for the binding process.

The interactions between transport proteins and newly obtained compounds are important for distribution and metabolism [73]. BSA was irradiated by 295 nm excitation wavelength, that corresponds to tryptophan residues in positions 134 and 213 nm. The measurements were performed after each addition of organotin compounds, and the fluorescence spectra are presented in Figure 7 and Figure S10 for measurements at three temperatures that mimic normal body temperature.

As shown in Figure 7 and Figure S10, upon the addition of organotin(IV) compounds, there was a decrease in the fluorescence intensity. A position of maxima was slightly shifted towards lower wavelengths, similar to the previous contribution [41]. These emission spectra changes prove that the investigated compounds bind in the active positions of BSA [74]. The decrease in intensity with increased concentration followed a double-log Stern–Volmer equation. High correlation coefficients were found for this dependency (higher than 0.997 (Table 3). The binding constants changed uniformly with temperature. In the case of ***n*Bu_3_SnL1**, their values were between 2.74 × 10^6^ (27 °C) and 9.23 × 10^4^ M^−1^ (27 °C). The numbers of binding positions were between 1.35 and 1.08, proving that one molecule was bound to one BSA molecule. A similar effect was found for other organotin(IV) compounds with hydrazone Schiff bases [74]. On the other hand, where the binding of ***n*Bu_3_SnL2** is concerned, the binding constants increased with temperature, between 2.68 × 10^5^ and 1.45 × 10^6^ M^−1^.

Different behaviors in the investigated temperature range led to different values of changes in the process’s enthalpy and entropy, namely −261.9 kJ mol^−1^ and −748.5 J mol^−1^ K^−1^ for the first compound, and 130.4 kJ mol^−1^ and 538.6 J mol^−1^ K^−1^ for the second. The first compound showed similar behavior to the organotin(IV) compound with three methyl groups [41] as the system’s entropy decreased, signifying that the free movement of the complex was limited by the presence of different amino acids in the active pocket. The negative change in entropy of the binding of ***n*Bu_3_SnL1** results from the stronger interactions within the active pocket than for two separate compounds. The binding of the second compound was characterized by the positive change in enthalpy and entropy due to the size of the compound and better fitting within the active pocket. These results prove that the absence of elongated delocalization within the ligand molecules’ aliphatic chain is important for binding to BSA. Both compounds bind spontaneously to the transport protein, as shown by the negative values of change in Gibbs free energy. For the first compound, spontaneity decreases with temperature, between −37.3 and −29.8 kJ mol^−1^, while it increases for the second, between −31.2 and −36.6 kJ mol^−1^. These interactions are important for applying the obtained compounds and their biological activity and distribution.

**Figure 8:**
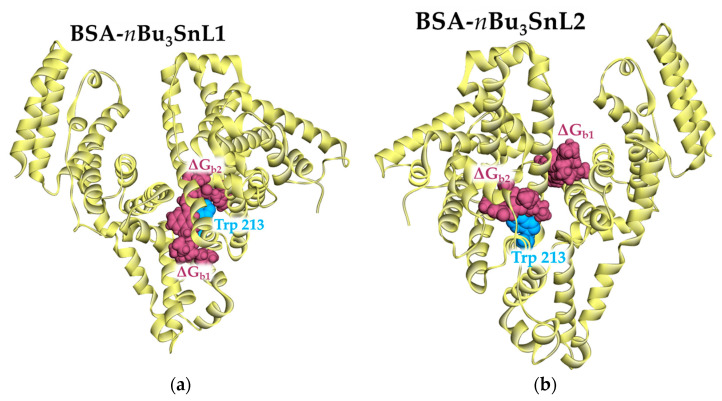
The most stable positions of (**a**) ***n*****Bu_3_SnL1** and (**b**) ***n*Bu_3_SnL2** compounds within BSA structure.

The interactions between amino acids and organotin(IV) compounds were analyzed by molecular docking simulations to explain the binding at the atomic level. The optimized structures were employed for the molecular docking simulations as flexible ligands. Upon simulation, two positions with the highest binding affinity (lowest ΔG_b_) for each compound were found, as presented in Figure 8. It is important to observe that these binding positions are located near a fluorescent amino acid (Trp213), which coincides with the observed decrease in the fluorescence intensity. The changes in Gibbs free energy of binding in the case of ***n*Bu_3_SnL1** are −38.5 (ΔG_b1_) and −31.4 kJ mol^−1^ (ΔG_b2_). The first value, calculated at 298 K, reproduces well the experimental value of −37.0 kJ mol^−1^ at 300 K. Conversely, two similar ΔG_b_ values were obtained for ***n*Bu_3_SnL2**, namely ΔG_b1_ = −28.5 kJ mol^−1^ and ΔG_b2_ = −28.3 kJ mol^−1^. The second value represents the binding process near Trp213, and it can be concluded that this position describes the experimental results. The obtained value is also very close to the experimentally determined one (−31.0 kJ mol^−1^ at 300 K). The differences between experimental and theoretical values of 1.5 and 2.5 kJ mol^−1^ result from the simulation procedure involving crystallographic protein structure, which slightly changes when water molecules and ions in body fluids surround the molecule. Similar differences were found when trimethyltin(IV) compound bearing 3-(4-methyl-2-oxoquinolin-1(2H)-yl)propanoate ligand was examined.

**Figure 9:**
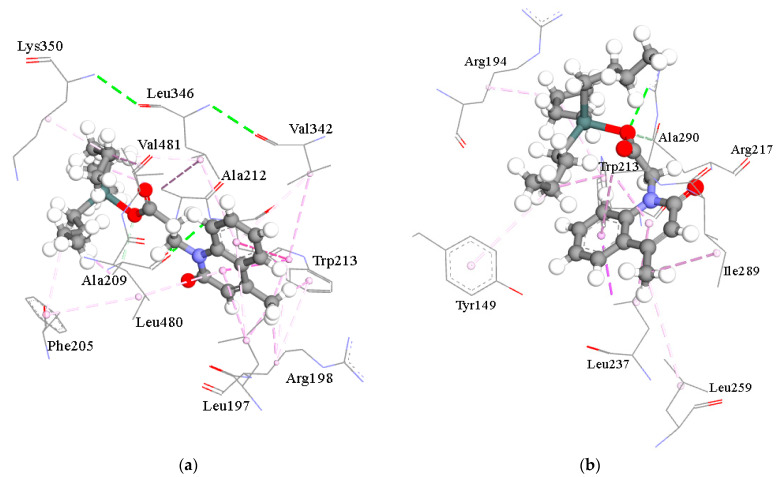
The most important interactions between organotin(IV) compounds (**a**) ***n*****Bu_3_SnL1** and (**b**) ***n*Bu_3_SnL2** and BSA in the active pockets close to Trp213. Different colors of the interactions represent the following: van der Waal interactions and carbon–hydrogen bonds—light green; conventional hydrogen bonds—dark green; π–alkyl—orange; π–π stacked—dark pink; alkyl and π–alkyl—light pink (Hydrogen—white, carbon—gray, nitrogen—blue, oxygen—red, tin—dark green).

The interactions between compounds and active pockets in the BSA structure are depicted in Figure 9. Multiple interactions contribute to the binding energy. Where ***n*Bu_3_SnL1** is concerned, weak hydrogen bonds are present between the hydrogen atom of butyl moiety and Arg208 and between the oxygen atom and Ala209. The interaction with Trp213 is denoted as π–π stacking, and the change in the chemical environment of this amino acid is the reason for the decrease in fluorescence, as experimentally observed. The most numerous interactions are van der Waal interactions with Ser201, Leu210, Asp323, Arg194, and others. Although weak, these interactions influence the structure of the protein. Aromatic rings in organotin(IV) structures are responsible for π–alkyl interactions with Leu197, Val342, Leu480, and Leu346. Alkyl interactions with surrounding amino acids are formed through butyl moieties. This analysis proves the importance of rigid conjugated rings and flexible butyl groups in interactions with proteins. The interactions between ***n*Bu_3_SnL2** include one conventional hydrogen bond between carboxyl oxygen and Arg217 and a weak hydrogen bond with Arg198. The second organotin(IV) is also close to Trp213 and a weak π–alkyl interaction is observed. Again, the most numerous are van der Waal interactions, with Phe222, Ile263, Ser286, Val240, and others. Ile289, Leu259, Tyr149, Arg194, and Ala290 interact with ***n*Bu_3_SnL2** through alkyl and π–alkyl interactions. There is an additional contribution to the binding energy through π–σ contact with Leu237. When protein affinities of both compounds are compared (−38.5 vs. −28.5 kJ mol^−1^), it can be concluded that the flexibility of the aliphatic chain attached to tin(IV), through the additional CH_2_ group, is the main reason for the increased number of interactions between butyl moieties of ***n*Bu_3_SnL1** and surrounding amino acids. The rest of the interactions are similar for both compounds.

## 4. Conclusions

Two novel tributyltin(IV) compounds were successfully synthesized and thoroughly characterized using a range of standard analytical techniques. Following the data collected by FT-IR and NMR spectroscopy, the carboxylato ligands, **L1^−^** and **L2^−^**, coordinate to the tributyltin(IV) moiety via the oxygen atom of the carboxylic group. The ^119^Sn NMR spectroscopy confirmed a four-coordinated tetrahedral environment around the tin atom in a solution of aprotic solvents. The crystal structure of ***n*Bu_3_SnL2** confirmed the monodentate coordination mode of the carboxylic group with the tributyltin(IV) moiety. However, due to the coordination of an oxygen atom from the quinolone ring of the adjacent molecule, the formation of a polymeric *zig*-*zag* chain was observed, confirming that this compound is five-coordinated in the solid state. This study demonstrates that the organotin moiety significantly influences the mechanism of cell death, emphasizing that not solely L, in this case carboxylato *N*-functionalized 2-quinolone, dictates the outcome. ***n*Bu_3_SnL1** and ***n*Bu_3_SnL2** induce apoptosis initiated by cell detachment and a moderate enhancement of ROS/RNS production, whereas the compounds with Me or Ph moieties employ different mechanisms. These findings also highlight the importance of the organotin component of the compounds in defining their anticancer efficacy and underscore the need for further research to fully understand these mechanisms and their potential for therapeutic applications.

## Figures and Tables

**Table 1:** Experimental and theoretical ^1^H and ^13^C NMR chemical shifts of ***n*Bu_3_SnL1** (at B3LYP-D3BJ/6-311++G(d,p)(H,C,N,O)/def2-TZVP(Sn) level of theory).

^1^H			^13^C		
H atom	Exp. [ppm]	Calc. [ppm]	C atom	Exp. [ppm]	Calc. [ppm]
H^6^	7.26	7.38	CH_3_	19.04	21.55
H^8^	7.49	8.12	C^12^	32.84	37.96
H^7^	7.57	7.76	C^13^	38.81	43.27
H^5^	7.72	7.90	C^8^	114.34	114.68
H^3^	6.59	6.49	C^3^	121.10	118.68
H^13^	4.58	3.71	C^10^	121.68	121.40
H^12^	2.74	3.17	C^6^	121.82	120.60
CH_3_	2.47	2.42	C^5^	125.4	125.04
H^α^	1.63	1.11	C^7^	130.52	129.92
H^β^	1.37	1.40	C^9^	138.80	138.14
H^γ^	1.29	1.26	C^4^	146.48	148.94
H^δ^	0.93	0.84	C2	161.71	158.41
R		0.98	C^11^	176.30	174.66
MAE [ppm]		0.33	C^δ^	13.66	15.64
			C^α^	16.51	19.55
			C^γ^	27.04	31.85
			C^β^	27.83	31.35
			R		1.00
			MAE [ppm]		2.28

**Table 2:** IC_50_ concentrations [µM]* determined after 72 h of action of ***n*Bu_3_SnL1** and ***n*Bu_3_SnL2** as well as related organotin(IV) compounds, ligand precursors (**HL1** and **HL2**) and cisplatin.

Compound	Assays	MCF-7	A375	HCT116	4T1	B16	CT26
**HL1**, **HL2** *	MTT [µM]	>200					
	CV [µM]	>200					
**Me_3_SnL1** **	MTT [µM]	5.7 ± 0.2	5.4 ± 0.5	6.2 ± 0.4	17.2 ± 2.4	8.1 ± 0.15	11.9 ± 1.5
	CV [µM]	11.4 ± 2.7	10.9 ± 1.9	8.0 ± 0.4	18.9 ± 1.8	10.6 ± 1.0	16.1 ± 2.0
***n*** **Bu_3_SnL1**	MTT [µM]	0.50 ± 0.05	0.50 ± 0.10	0.50± 0.05	0.25 ± 0.04	0.20 ± 0.01	0.22 ± 0.00
	CV [µM]	0.60 ± 0.10	0.70 ± 0.05	0.80 ± 0.00	0.30 ± 0.00	0.20 ± 0.06	0.30 ± 0.04
***n*** **Bu_3_SnL2**	MTT [µM]	0.30 ± 0.02	0.31 ± 0.01	0.30 ± 0.02	0.22 ± 0.02	0.20 ± 0.02	0.24 ± 0.05
	CV [µM]	0.50 ± 0.10	0.60 ± 0.05	0.55 ± 0.0.5	0.29 ± 0.02	0.20 ± 0.05	0.30 ± 0.00
**Ph_2_Sn(L1)_2_** *	MTT [µM]	0.10 ± 0.00	0.30 ± 0.05	0.15 ± 0.02	0.30 ± 0.03	0.36 ± 0.01	0.30 ± 0.01
	CV [µM]	0.20 ± 0.02	0.36 ± 0.05	0.26 ± 0.02	0.40 ± 0.01	0.39 ± 0.07	0.40 ± 0.07
**Ph_2_Sn(L2)_2_** *	MTT [µM]	0.60 ± 0.03	0.60 ± 0.05	0.60 ± 0.03	0.50 ± 0.02	0.50 ± 0.10	0.70 ± 0.04
	CV [µM]	0.60 ± 0.02	0.62 ± 0.02	0.46 ± 0.00	0.70 ± 0.00	0.50 ± 0.20	0.80 ± 0.02
**Ph_3_SnL1** ***	MTT [µM]	0.10 ± 0.03	0.10 ± 0.02	0.10 ± 0.01	0.10 ± 0.01	0.10 ± 0.03	0.16 ± 0.02
	CV [µM]	0.15 ± 0.03	0.12 ± 0.00	0.13 ± 0.03	0.17 ± 0.01	0.10 ± 0.04	0.18 ± 0.01
**Ph_3_SnL2** ***	MTT [µM]	0.10 ± 0.01	0.10 ± 0.01	0.05 ± 0.03	0.10 ± 0.01	0.10 ± 0.01	0.10 ± 0.01
	CV [µM]	0.10 ± 0.00	0.12 ± 0.01	0.10 ± 0.01	0.10 ± 0.01	0.10 ± 0.02	0.10 ± 0.02
**Cisplatin** *	MTT [µM]	5.00 ± 0.70	8.10 ± 0.70	5.00 ± 2.30	1.60 ± 0.20	8.30 ± 1.60	4.30 ± 0.20
	CV [µM]	12.60 ± 2.50	10.70 ± 1.60	12.30 ± 3.50	2.50 ± 0.10	18.00 ± 1.80	4.70 ± 0.40

* mean ± SD; * [39]; ** [41]; *** [40].

**Table 3:** Binding process parameters for the interaction between obtained complexes and BSA.

Compound	T [K]	K_b_ [M^−1^]	n	R^2^	ΔH_b_ [kJ mol^−1^]	ΔS_b_ [J mol^−1^ K^−1^]	ΔG_b_ [kJ mol^−1^]
***n*Bu_3_SnL1**	300	2.74 × 10^6^	1.35	0.999	−261.9	−748.5	−37.3
	308	7.53 × 10^5^	1.24	0.997			−33.6
	310	9.23 × 10^4^	1.08	0.999			−29.8
***n*Bu_3_SnL2**	300	2.68 × 10^5^	1.204	0.999	130.4	538.6	−31.2
	308	6.67 × 10^5^	1.24	0.997			−33.9
	310	1.45 × 10^6^	1.45	0.999			−36.6

## Data Availability

Data supporting obtained results can be obtained from the authors upon request.

## Supplementary Materials

The following supporting information can be downloaded at: https://www.mdpi.com/article/10.3390/pharmaceutics16121529/s1, Table S1: Crystal data and structure refinement for ***n*Bu_3_SnL2**; Table S2: Bond lengths and angles for the compound ***n*Bu_3_SnL2**; Table S3: DFT/B3LYP-D3BJ/6-311+G(d,p)/def2-TZVPD optimized bond and angles (in °) of ***n*Bu_3_SnL1** and ***n*Bu_3_SnL2**; Table S4: Experimental and theoretical ^1^H and ^13^C NMR chemical shifts of ***n*****Bu_3_SnL2**; Figure S1: ORTEP diagram (25% probability ellipsoids) of ***n*Bu_3_SnL2** in the solid state showing split occupancies of two *n*Bu groups 0.58/0.42 (C14–C18) and 0.38/0.62 (C22–C25) Figure S2: FT-IR spectra of the tributyltin(IV) complexes: (a) ***n*Bu_3_SnL1** and (b) ***n*Bu_3_SnL2**; Figure S3: NMR spectra of the tributyltin(IV) complex ***n*Bu_3_SnL1**: (a) ^1^H; (b) ^13^C; (c) ^119^Sn; Figure S4: NMR spectra of the tributyltin(IV) complex ***n*Bu_3_SnL2**: (a) ^1^H; (b) ^13^C; (c) ^119^Sn; Figure S5: Selected BCPs for the optimized structures (at B3LYP-D3BJ/6-311++G(d,p)(H,C,N,O)/def2-TZVP(Sn) level of theory) of investigated compounds; Figure S6: UV-Vis spectra of tributyltin(IV) complexes in water/DMSO solution, immediately after dissolution and after 24, 48 and 72 h for compounds: (a) ***n*Bu_3_SnL1** and (b) ***n*Bu_3_SnL2**; Figure S7: Compound ***n*Bu_3_SnL1** decreased the viability of all cancer cells. Cells were exposed to a wide range of concentration of selected compound. After 72 h, MTT and CV tests were performed; Figure S8: Compound ***n*Bu_3_SnL2** decreased the viability of all cancer cells. Cells were exposed to a wide range of concentration of selected compound. After 72 h, MTT and CV tests were performed; Figure S9: Compounds ***n*Bu_3_SnL1** and ***n*Bu_3_SnL2** showed no effect on the viability of primary peritoneal exudate cells (PEC). Cells were exposed to experimental drugs and after 72 h CV test was performed; Figure S10: Fluorescence emission spectra of BSA for the titration with ***n*Bu_3_SnL1** at (a) 27°, (b) 32°, (c) 37°, and (d) Van ’t Hoff plot for the binding process.
