# Supplementary material for: Organic Moiety on Sn(IV) Does Matter for In Vitro Mode of Action: nBu3Sn(IV) Compounds with Carboxylato N-Functionalized 2-Quinolones Induce Anoikis-like Cell Death in A375 Cells
Source: Pharmaceutics. 2024 Nov 28;16(12):1529. doi: 10.3390/pharmaceutics16121529 (PMC11679857; doi:10.3390/pharmaceutics16121529)
Supplement: Supplementary file 1 [file pharmaceutics-16-01529-s001.zip › pharmaceutics-3264233-supplementary.pdf]

Supplementary Materials for paper:

Organic Moiety on Sn(IV) Does Matter for In Vitro Mode of Action: *n*Bu$_3$Sn(IV) Compounds with Carboxylato *N*-Functionalized 2-Quinolones Induce Anoikis-like Cell Death in A375 Cells

Marijana P. Kasalović [1,2], Sanja Jelača [3], Dušan Dimić [4], Danijela Maksimović-Ivanić [3], Verica V. Jevtić [2], Sanja Mijatović [3], Tobias Rüffer [5], Goran N. Kaluđerović [1,*] and Nebojša Đ. Pantelić [6,*]

[1] Department of Engineering and Natural Sciences, University of Applied Sciences Merseburg, Eberhard-Leibnitz-Straße 2, 06217 Merseburg, Germany

[2] Department of Chemistry, Faculty of Science, University of Kragujevac, Radoja Domanovića 12, 34000 Kragujevac, Serbia

[3] Department of Immunology, Institute for Biological Research "Siniša Stanković"—National Institute of the Republic of Serbia, University of Belgrade, Bulevar Despota Stefana 142, 11060 Belgrade, Serbia

[4] Faculty of Physical Chemistry, University of Belgrade, Studentski Trg 12–16, 11000 Belgrade, Serbia

[5] Institute of Chemistry, Chemnitz University of Technology, Straße der Nationen 62, 09111 Chemnitz, Germany

[6] Department of Chemistry and Biochemistry, Faculty of Agriculture, University of Belgrade, Nemanjina 6, 11080 Belgrade, Serbia

* Correspondence: goran.kaluderovic@hs-merseburg.de (G.N.K.); pantelic@agrif.bg.ac.rs (N.Đ.P.)

TABLE OF CONTENTS OF SUPPLEMENTARY MATERIAL

This supplementary material contains:

1) Crystal data and structure refinement for ***n*****Bu$_3$SnL2** (Table S1)
2) Bond lengths and angles for the compound ***n*****Bu$_3$SnL2** (Table S2)
3) DFT/B3LYP-D3BJ/6-311+G(d,p)/ def2- TZVPD optimized bond and angles (in °) of ***n*****Bu$_3$SnL1** and ***n*****Bu$_3$SnL2** (Table S3)
4) Experimental and theoretical ^{1}H and ^{13}C NMR chemical shifts of ***n*****Bu$_3$SnL2** (Table S4)
5) ORTEP diagram (25 % probability ellipsoids) of ***n*****Bu$_3$SnL2** in the solid state showing split occupancies of two *n*Bu groups 0.58/0.42 (C14–C18) and 0.38/0.62 (C22–C25) (Figure S1)
6) FT-IR spectra of tributyltin(IV) complexes ***n*****Bu$_3$SnL1** and ***n*****Bu$_3$SnL2** (Figure S2)
7) ^{1}H, ^{13}C NMR and ^{119}Sn NMR spectra of tributyltin(IV) complexes (Figures S3 & S4)
8) Selected BCPs for the optimized structures of ***n*****Bu$_3$SnL1** and ***n*****Bu$_3$SnL2** (Figure S5)
9) UV-Vis spectra of tributyltin(IV) complexes in water/DMSO solution, immediately after dissolution and after 24, 48 and 72 hours for compounds: a) ***n*****Bu$_3$SnL1** and b) ***n*****Bu$_3$SnL2** (Figure S6)
10) Cell viability (Figures S7–S9)
11) Fluorescence emission spectra of BSA for the titration with ***n*****Bu$_3$SnL1** (Figure S10)

Table S1. Crystal data and structure refinement for ***n*Bu$_3$SnL2**

Empirical formula	***n*Bu$_3$SnL2** ($C_{24}H_{37}NO_3Sn$)
**Formula weight**	506.23
**Temperature**	200 K
**Radiation**	Mo Kα
**Wavelength**	0.71073 Å
**Crystal system**	monoclinic
**Space group**	$P2_1/n$
a	9.9304(4) Å
b	14.6622(4) Å
c	17.8810(5) Å
**α, β, γ (°)**	90°, 105.444(3)°, 90°
**Volume**	2509.49(15) Å^3
Z	4
**Calculated density**	1.340 mg/mm^3
**Absorption coefficient**	1.040 mm^{-1}
***F*(000)**	1048
**Crystal size**	0.5 × 0.5 × 0.4 mm
**θ range for data collection**	3.032 to 28.417°
**Limiting indices**	$-13 \leq h \leq 12$, $-19 \leq k \leq 19$, $-23 \leq l \leq 23$
**Reflections collected**	36990
**Independent reflections**	13355 [R_{int} = 0.0260, R_{sigma} = 0.0199]
**Completeness to θ = 25.242**	99.8%
**Absorption correction**	Semi-empirical from equivalents
**Max. and min. transmission**	1.00000 and 0.94862
**Refinement method**	Full-matrix least squares on F^2
**Data / restraints / parameters**	13355/ 132 / 337
**Goodness-of-fit on F^2**	1.071
**Final *R* indices [$I \geq 2\sigma(I)$]**	R_1 = 0.0360, wR_2 = 0.0983
***R* indices (all data)**	R_1 = 0.0488, wR_2 = 0.1025
**Extinction coefficient**	n/a
**Largest diff. peak and hole**	0.481 and -0.733 e·A^{-3}

Table S2. Selected bond lengths (Å) and angles (°) of ***n*****Bu$_3$SnL2**

**Bond Lengths** (Å)		**Bond Angle** (°)	
N1–C2	1.366(3)	N1–C2–O3	119.4(2)
C2–C3	1.425(4)	N1–C2–C3	117.5(2)
C11–O3	1.267(3)	O2–C2–O3	126.9(3)
C3–C4	1.352(4)	C2–C3–C4	123.4(2)
C4-C13	1.486(4)	C3–C4–C10	118.0(2)
C5–C6	1.368(5)	C3–C4–C13	121.4(3)
C6–C7	1.378(5)	C4–C10–C5	121.7(3)
C7–C8	1.375(4)	C5–C6–C7	119.7(3)
C8–C9	1.400(4)	C6–C7–C8	121.5(3)
C9–C10	1.405(4)	C8–C9–N1	120.8(2)
C9–N1	1.392(3)	C8–C9–C10	119.3(2)
C10–C4	1.440(4)	N1–C12–C11	113.7(2)
C10–C5	1.409(4)	C12–C11–O3	114.6(2)
N1–C12	1.460(3)	C12–C11–O2	118.5(2)
C12–C11	1.523(4)	O3–C11–O2	126.9(3)
C2–O1	1.254(3)	C11–O3–Sn1A	128.51(17)
C11–O2	1.216(3)	C2–O1–Sn1	138.97(19)
Sn1–C14	2.132(10)	O1–Sn1–O3B	170.67(8)
C14–C15	1.479(16)	C18–Sn1–C14	126.4(3)
C15–C16	1.583(15)	C18–Sn1–C22	107.7(2)
C16–C17	1.43(2)	C14–Sn1–C22	124.8(4)
Sn1–C18	2.120(3)	O1–Sn1–C14	87.2(4)
C18–C19	1.514(4)	O3B–Sn1–C14	96.9(4)
C19–C20	1.520(4)	O1–Sn1–C18	87.54(10)
C20–C21	1.507(5)	O3B–Sn1–C18	96.69(10)
Sn1–C22	2.131(11)	O1–Sn1–C22	85.3(3)
C22–C23	1.162(13)	O3B–Sn1–C22	85.5(3)
C23–C24	1.497(14)	Sn1–C14–C15	114.7(6)
C24–C25	1.39(4)	C14–C15–C16	119.8(10)
Sn1–O3A	2.1533(18)	C15–C16–C17	111.2(13)
		Sn1–C18–C19	113.4(2)
		C18–C19–C20	112.6(3)
		C19–C20–C21	113.2(3)
		Sn1–C22–C23	135.7(10)
		C22–C23–C24	133.8(11)
		C23–C24–C25	112.1(15)

Symmetry transformations: "A" = -*x*+ ½,*y*+ ½,-*z*+ ½; "B"= -x+ ½,y- ½,-z+ ½.

Table S3. The calculated Bond Critical Point (BCP) properties at the DFT/B3LYP-D3BJ/6-311+G(d,p)/ def2- TZVPD level of theory: the electron density (ϱ(r)) and its Laplacian value (∇^2ϱ(r)); the Lagrangian kinetic electron density (G(r)) and the potential electron density (V(r)); the density of the total energy of electrons (H(r)) and the Cremer–Kraka electronic energy density; the interatomic bond energy, E_{bond}.

	ϱ(r) [a.u.]	∇^2ϱ(r) [a.u.]	G(r) [kJ mol^{-1}]	V(r) [kJ mol^{-1}]	H(r) [kJ mol^{-1}]	-G(r)/V(r)	E_{bond} [kJ mol^{-1}]
***n*Bu$_3$SnL2**							
BCP1	0.083	0.326	251.9	-289.8	-37.9	0.87	-144.9
BCP2	0.106	0.074	168.4	-288.2	-119.8	0.58	-144.1
BCP3	0.106	0.078	171.8	-292.1	-120.3	0.59	-146.1
BCP4	0.106	0.075	170.2	-291.4	-121.2	0.58	-145.7
BCP1′	0.005	0.016	9.0	-7.3	1.7	1.23	-3.65
BCP2′	0.09	0.027	15.7	-13.7	2.0	1.15	-6.9
BCP1″	0.004	0.010	5.5	-4.2	1.3	1.31	-2.1
BCP2″	0.004	0.012	6.5	-4.9	1.6	1.33	-2.5
***n*Bu$_3$SnL1**							
BCP1	0.085	0.341charac	265.2	-306.4	-41.2	0.87	-153.2
BCP2	0.106	0.078	171.6	-291.8	-120.2	0.59	-145.9
BCP3	0.106	0.074	169.0	-289.4	-120.4	0.58	-144.7
BCP4	0.106	0.077	170.4	-290.5	-120.1	0.59	-145.3
BCP1′	0.007	0.021	12.3	-10.5	1.8	1.17	-5.3
BCP1″	0.003	0.010	5.4	-4.0	1.4	1.35	-2.0

Table S4. Experimental and theoretical (at B3LYP-D3BJ/6-311++G(d,p)(H,C,N,O)/def2-TZVP(Sn) level of theory) ^{1}H and ^{13}C NMR chemical shifts of ***n*Bu$_3$SnL2**.

**^{1}H**			**^{13}C**		
**H atom**	**Exp. [ppm]**	**Calc. [ppm]**	**C atom**	**Exp. [ppm]**	**Calc. [ppm]**
H^6	7.16	7.34	C^{12}	44.06	460.05
H^8	7.26	7.13	CH^3	19.09	21.65
H^7	7.51	7.67	C^8	114.24	113.54
H^5	7.72	7.88	C^3	120.78	118.52
H^3	6.63	6.59	C^{10}	121.5	120.94
H^{12}	5.1	4.98	C^6	121.97	120.86
CH^3	2.48	2.45	C^5	125.37	125.03
H^α	1.55	1.09	C^7	130.31	130.50
H^β	1.35	1.20	C^9	139.28	139.00
H^γ	1.22	1.13	C^4	140.03	149.38
H^δ	0.89	0.48	C^2	161.65	158.40
R		0.99	C^{11}	172.84	173.14
MAE [ppm]		0.29	C^δ	13.62	15.55
			C^α	16.07	20.54
			C^γ	26.95	31.78
			C^β	27.68	31.26
			R		1.00
			MAE [ppm]		1.92

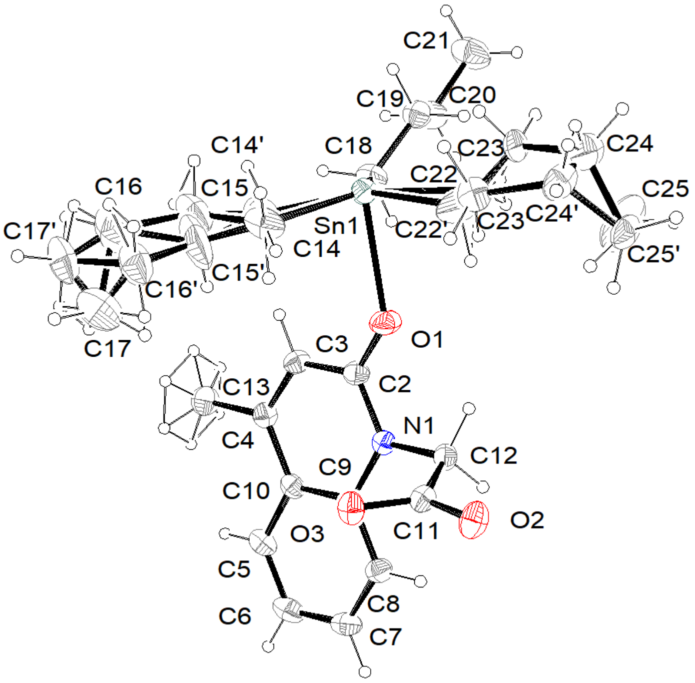

Figure S1. ORTEP diagram [42] (25 % probability ellipsoids) of ***n*Bu$_3$SnL2** in the solid state showing split occupancies of two *n*Bu groups 0.58/0.42 (C14–C18) and 0.38/0.62 (C22–C25)

a)

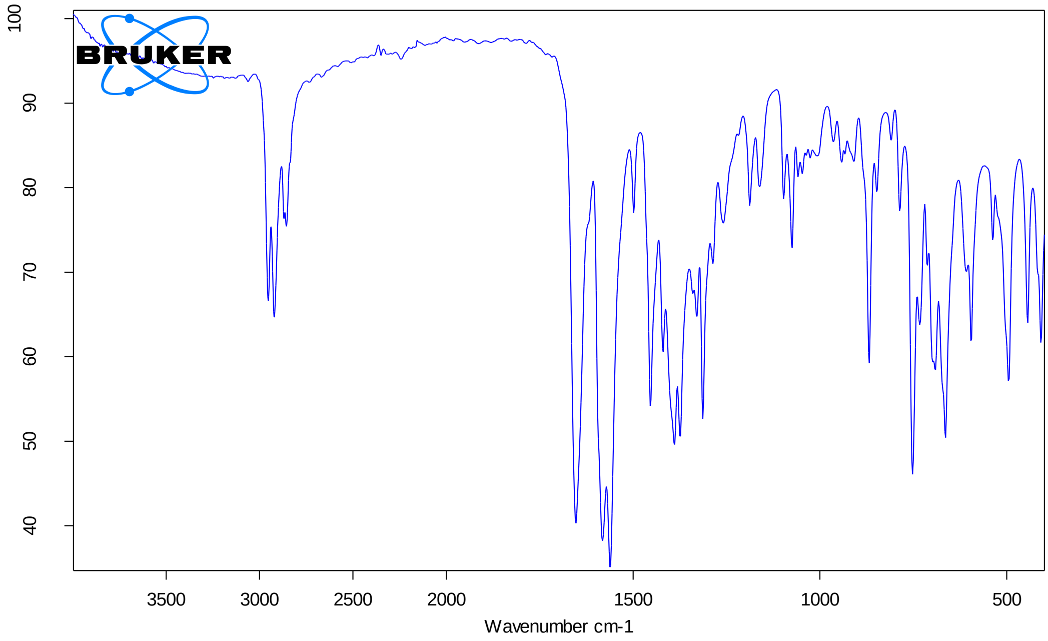

b)

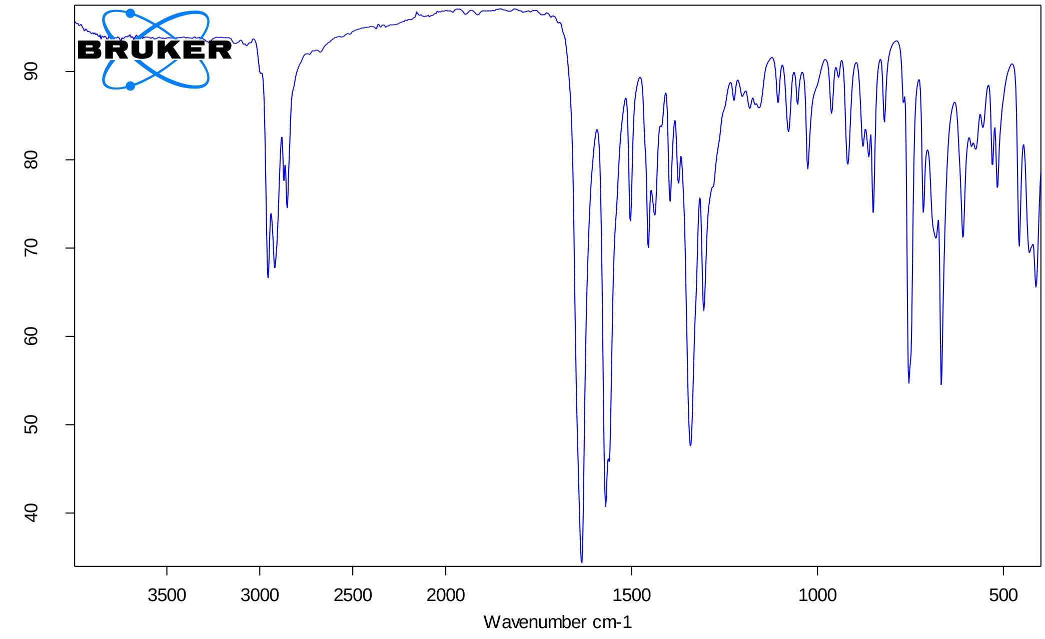

Figure S2. FT-IR spectra of the tributyltin(IV) complexes: a) ***n*Bu₃SnL1** and b) ***n*Bu₃SnL2**.

a)

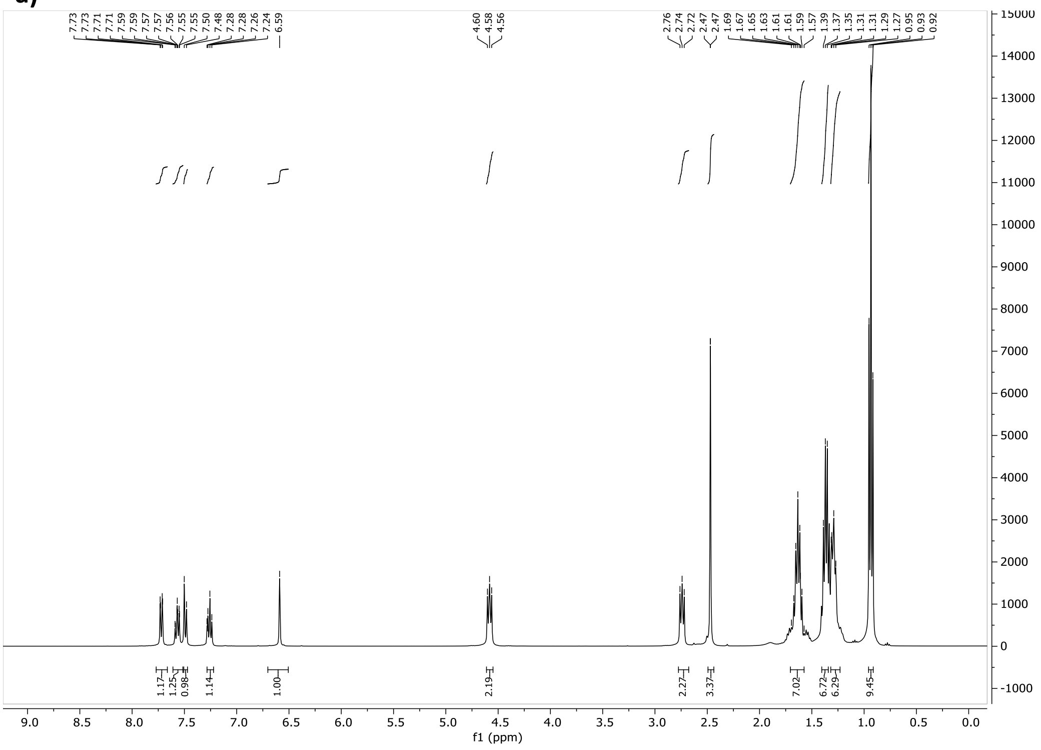
7.73 7.73 7.71 7.71 7.59 7.59 7.57 7.57 7.56 7.55 7.55 7.50 7.48 7.28 7.28 7.26 7.24 6.59
4.60 4.58 4.56
2.76 2.74 2.72 2.47 2.47 1.69 1.67 1.65 1.63 1.61 1.61 1.59 1.57 1.39 1.37 1.35 1.31 1.31 1.29 1.27 0.95 0.93 0.92
1.17 1.25 0.98 1.14 1.00 2.19 2.27 3.37 7.02 6.72 6.29 9.45
f1 (ppm)

b)

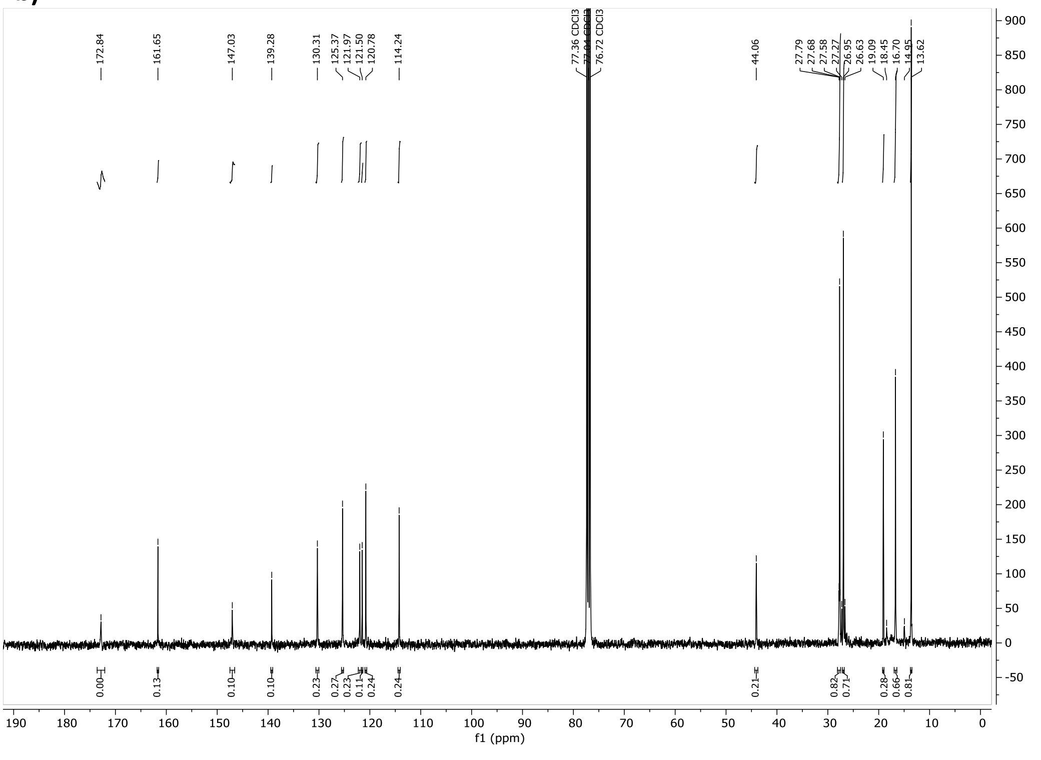
172.84
161.65
147.03
139.28
130.31 125.37 121.97 121.50 120.78
114.24
77.36 CDCl3
76.72 CDCl3
44.06
27.79 27.68 27.58 27.27 26.95 26.63 19.09 18.45 16.70 14.95 13.62
0.00 0.13 0.10 0.10 0.23 0.27 0.23 0.11 0.24 0.24 0.21 0.82 0.71 0.28 0.66 0.81
f1 (ppm)

c)

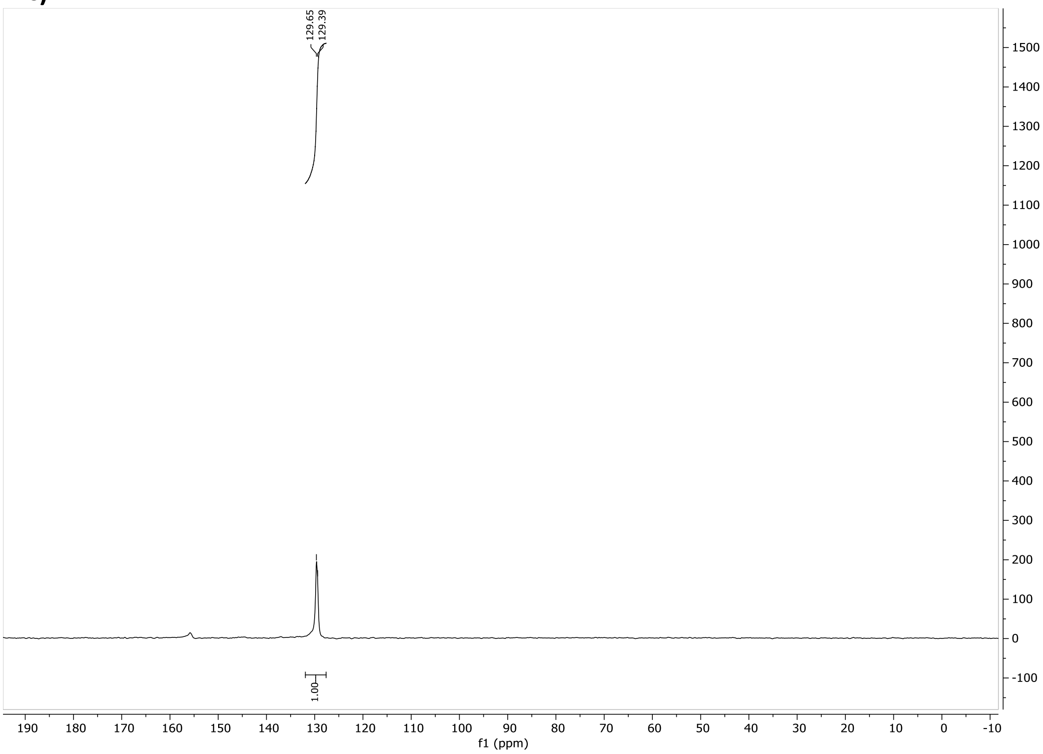

Figure S3. NMR spectra of the tributyltin(IV) complex ***n*Bu$_3$SnL1**: a) ^{1}H; b) ^{13}C; c) ^{119}Sn.

a)

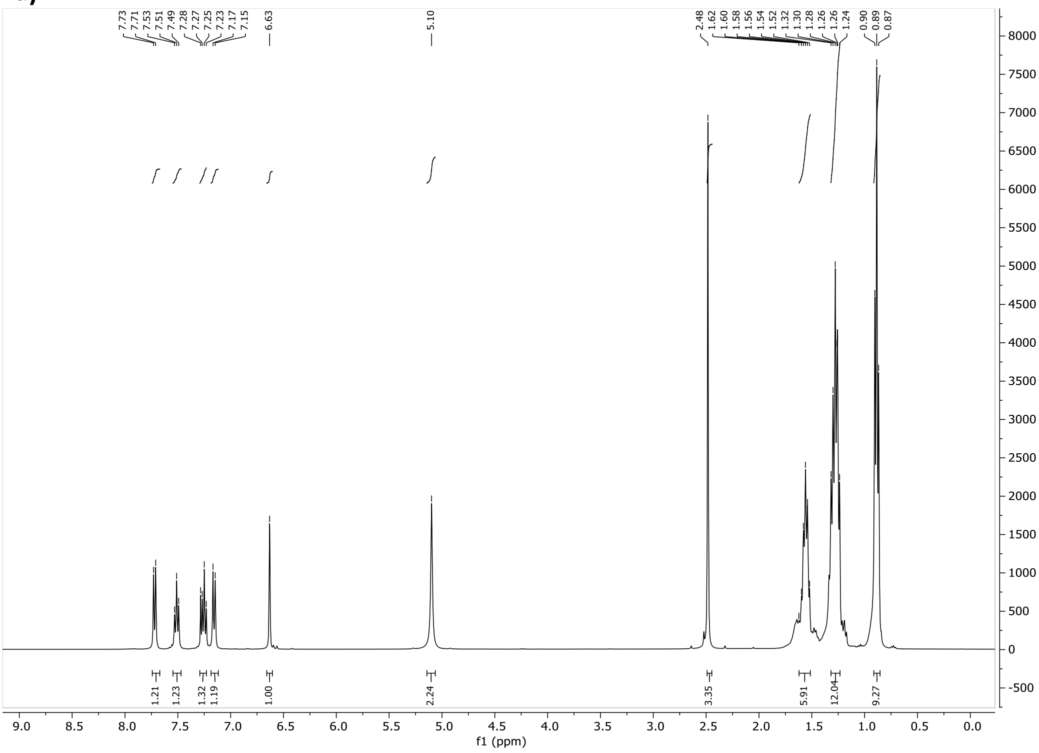
7.73
7.71
7.53
7.51
7.49
7.28
7.27
7.25
7.23
7.17
7.15
6.63
5.10
2.48
1.62
1.60
1.58
1.56
1.54
1.52
1.32
1.30
1.28
1.26
1.24
0.90
0.89
0.87
1.21
1.23
1.32
1.19
1.00
2.24
3.35
5.91
12.04
9.27
f1 (ppm)

b)

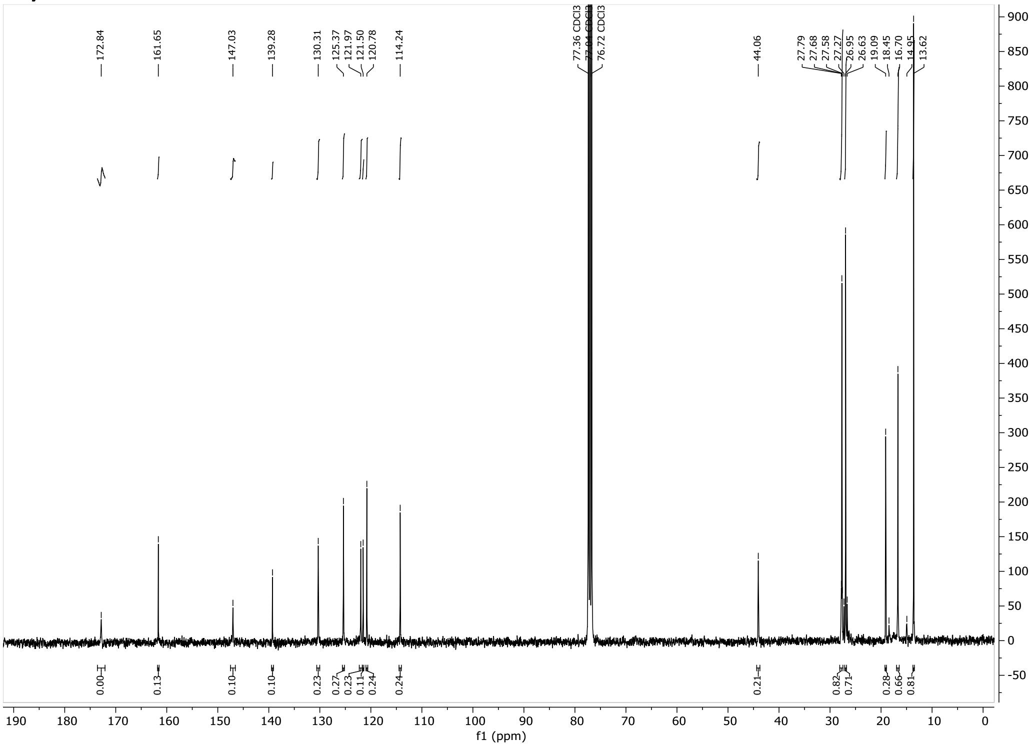
172.84
161.65
147.03
139.28
130.31
125.37
121.97
121.50
120.78
114.24
77.36 CDCl3
76.72 CDCl3
44.06
27.79
27.68
27.58
27.27
26.95
26.63
19.09
18.45
16.70
14.95
13.62
0.00
0.13
0.10
0.10
0.23
0.27
0.23
0.11
0.24
0.24
0.21
0.82
0.71
0.28
0.66
0.81
f1 (ppm)

c)

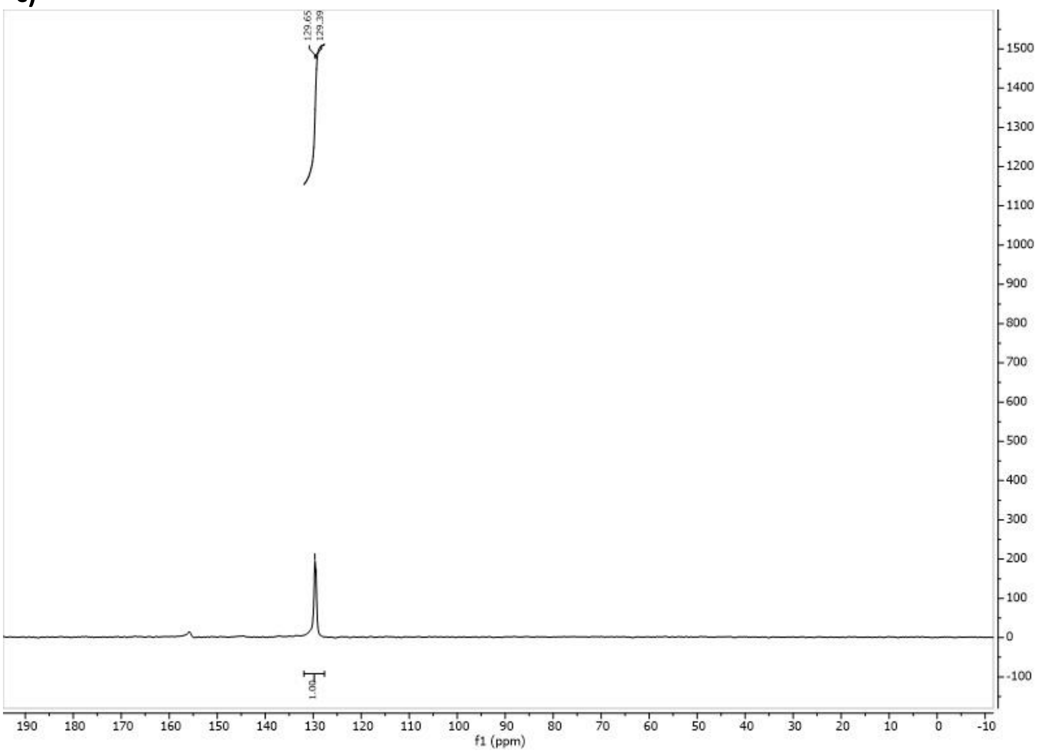

Figure S4. NMR spectra of the tributyltin(IV) complex ***n*Bu₃SnL2**: a) ^{1}H; b) ^{13}C; c) ^{119}Sn.

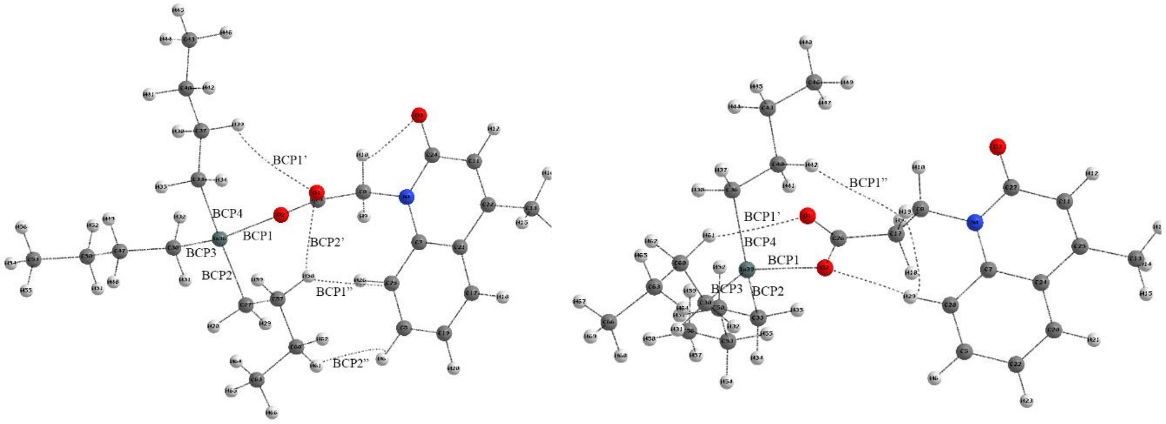

***n*Bu$_3$SnL1** ***n*Bu$_3$SnL2**

Figure S5. Selected BCPs for the optimized structures (at B3LYP-D3BJ/6-311++G(d,p)(H,C,N,O)/def2-TZVP(Sn) level of theory) of investigated compounds.

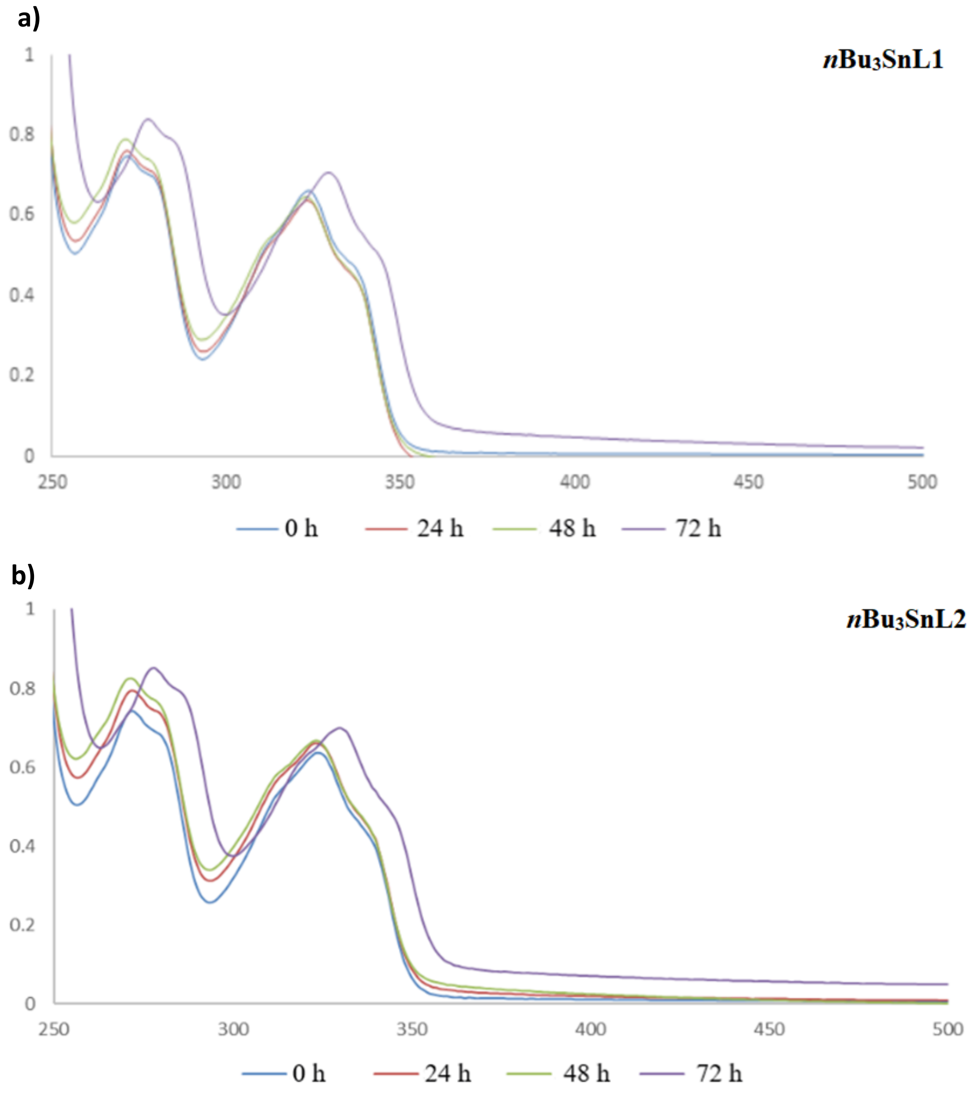

Figure S6. UV-Vis spectra of tributyltin(IV) complexes in water/DMSO solution, immediately after dissolution and after 24, 48 and 72 h for compounds: a) ***n*Bu$_3$SnL1** and b) ***n*Bu$_3$SnL2**.

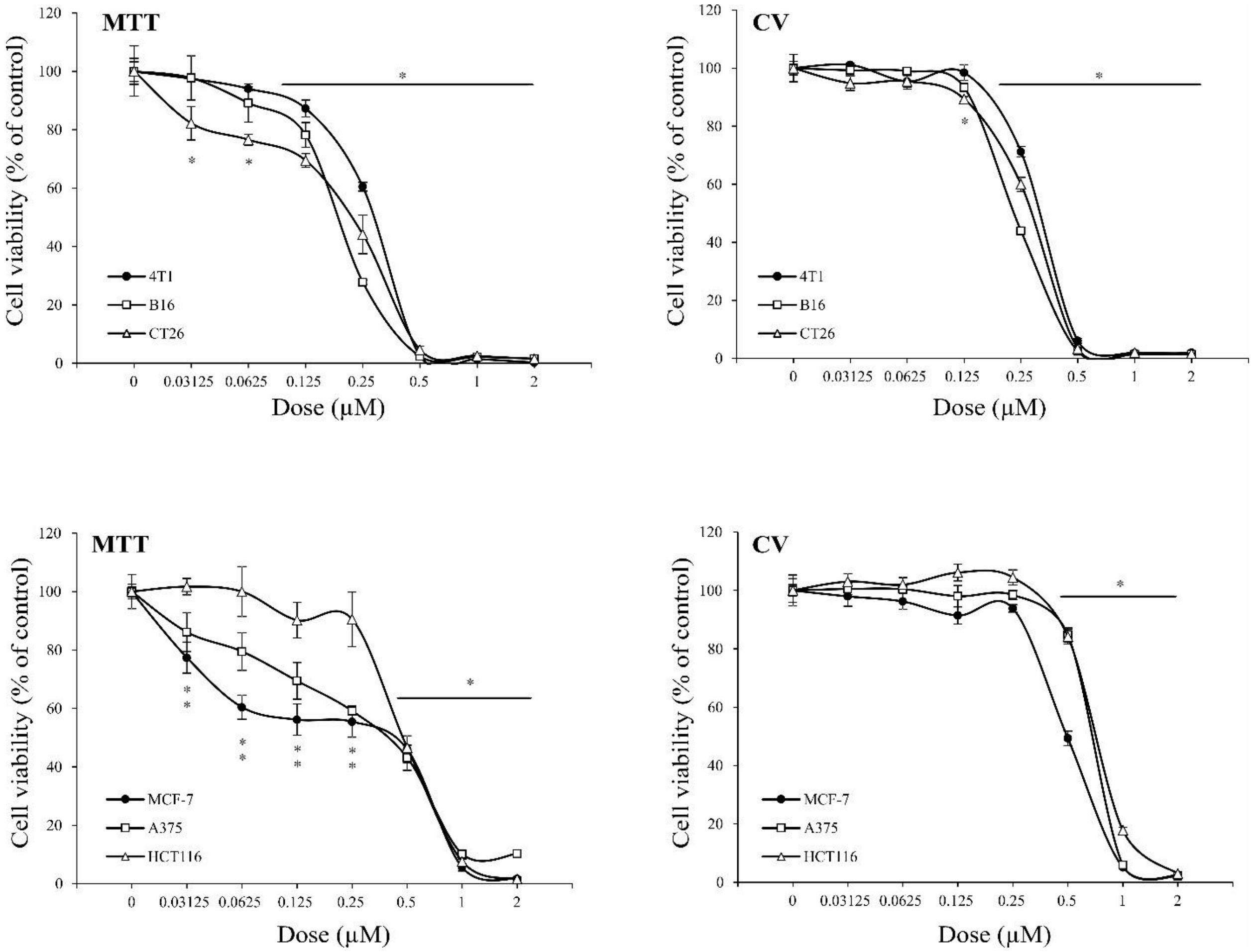

Figure S7. Compound ***n*Bu3SnL1** decreased the viability of all cancer cells. Cells were exposed to a wide range of concentration of the selected compound. After 72 h, MTT and CV tests were performed. All data are expressed as the mean ± SD from one representative replicate out of three independently repeated. Results are presented as a percentage of control (untreated cells with viability arbitrarily set as 100%). $^{*}p < 0.05$ is considered statistically significant.

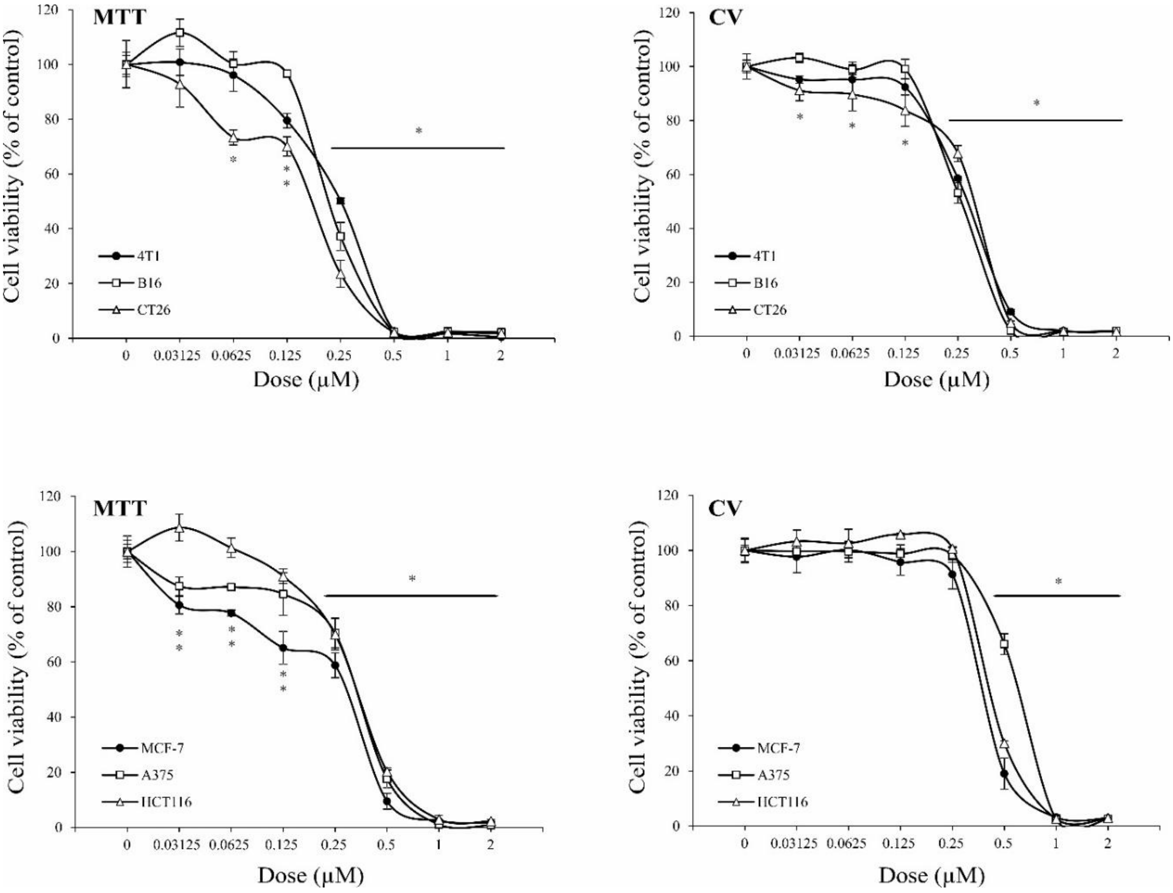

Figure S8. Compound ***n*Bu$_3$SnL2** decreased the viability of all cancer cells. Cells were exposed to a wide range of concentration of the selected compound. After 72 h, MTT and CV tests were performed. All data are expressed as the mean ± SD from one representative replicate out of three independently repeated. Results are presented as a percentage of control (untreated cells with viability arbitrarily set as 100%). *$p < 0.05$ is considered statistically significant.

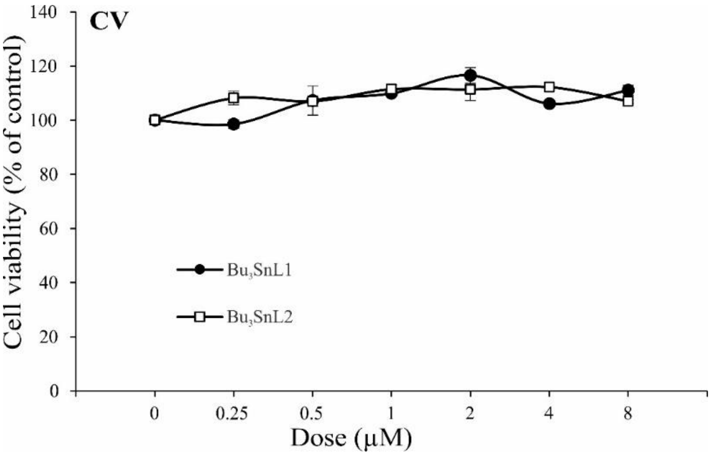

Figure S9. Compounds ***n*Bu$_3$SnL1** and ***n*Bu$_3$SnL2** showed no effect on the viability of primary peritoneal exudate cells (PEC). Cells were exposed to experimental drugs and after 72 h CV test was done. Data are expressed as the mean ± SD from one representative replicate out of three independently repeated. Results are presented as a percentage of control (untreated cells with viability arbitrarily set as 100%). *$p < 0.05$ is considered statistically significant.

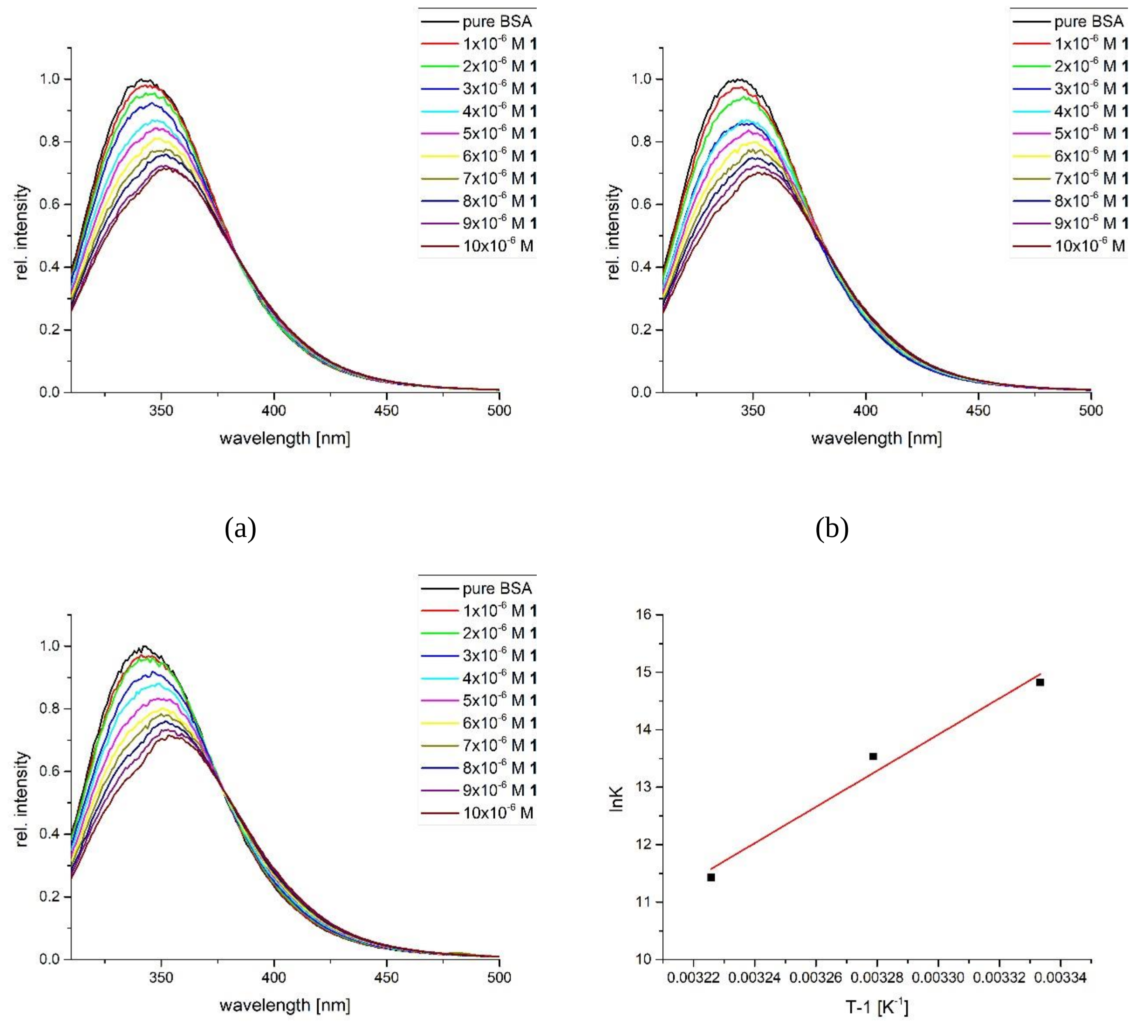

(a) (b)

(c) (d)

Figure S10. Fluorescence emission spectra of BSA for the titration with ***n*Bu$_3$SnL1** at (a) 27°, (b) 32°, (c) 37°, and (d) Van 't Hoff plot for the binding process

Reference:

42. Farrugia, L.J. *WinGX* and *ORTEP for Windows* : An Update. *J. Appl. Crystallogr.* **2012**, *45*, 849–854, doi:10.1107/S0021889812029111.
